# TGF-β1 increases permeability of ciliated airway epithelia via redistribution of claudin 3 from tight junction into cell nuclei

**DOI:** 10.1007/s00424-020-02501-2

**Published:** 2021-01-02

**Authors:** Carolin Schilpp, Robin Lochbaum, Peter Braubach, Danny Jonigk, Manfred Frick, Paul Dietl, Oliver H. Wittekindt

**Affiliations:** 1 Institute of General Physiology, Ulm University, Albert-Einstein-Allee 11, 89081 Ulm, Germany; 2 Institute of Pathology, Hannover Medical School, Carl-Neuberg-Str. 1, 30625 Hannover, Germany

**Keywords:** Lung, Tight junction, Claudin 3, TGF-β1, Motile cilia

## Abstract

TGF-β1 is a major mediator of airway tissue remodelling during atopic asthma and affects tight junctions (TJs) of airway epithelia. However, its impact on TJs of ciliated epithelia is sparsely investigated. Herein we elaborated effects of TGF-β1 on TJs of primary human bronchial epithelial cells. We demonstrate that TGF-β1 activates TGF-β1 receptors TGFBR1 and TGFBR2 resulting in ALK5-mediated phosphorylation of SMAD2. We observed that TGFBR1 and -R2 localize specifically on motile cilia. TGF-β1 activated accumulation of phosphorylated SMAD2 (pSMAD2-C) at centrioles of motile cilia and at cell nuclei. This triggered an increase in paracellular permeability via cellular redistribution of claudin 3 (CLDN3) from TJs into cell nuclei followed by disruption of epithelial integrity and formation of epithelial lesions. Only ciliated cells express TGF-β1 receptors; however, nuclear accumulations of pSMAD2-C and CLDN3 redistribution were observed with similar time course in ciliated and non-ciliated cells. In summary, we demonstrate a role of motile cilia in TGF-β1 sensing and showed that TGF-β1 disturbs TJ permeability of conductive airway epithelia by redistributing CLDN3 from TJs into cell nuclei. We conclude that the observed effects contribute to loss of epithelial integrity during atopic asthma.

**Supplementary Information:**

The online version contains supplementary material available at 10.1007/s00424-020-02501-2.

## Introduction

Enrichment of eosinophils is commonly observed in lungs of patients with atopic asthma [3] and contributes substantially to airway remodelling [46]. Eosinophils have been identified as a major source of TGF-β1 in the lung [73]. Eosinophil-derived TGF-β1 was identified as a major factor that triggers tissue remodelling in atopic asthma [39].

Transforming growth factors β (TGF-β) constitute a subgroup of the transforming growth factor superfamily. Three TGF-β isoforms have been identified so far, TGF-β1, -β2 and -β3 [42]. All members of transforming growth factor superfamily are synthesized as pre-pro-peptides that consist of a short N-terminal signalling peptide, a latency-associated protein (LAP) domain and the mature TGF domain [42]. After post-translational processing, TGF-β1 peptides are released from cells as latent TGF-β1 complexes (L-TGF-β1) [55] and are linked to extracellular matrix by L-TGF-β1 binding protein (LTBP) [4]. While the LAP peptides confer latency, LTBP is needed to release active TGF-β1 ligand from the L-TGF-β1 complex upon appropriate stimuli [4]. After being released from the latency complex, the active ligand TGF-β1 binds to its receptor (well summarized in [68]). Two receptor subunits are known, receptor type I and type II subunits. TGF-β1 binding to either subunit initiates their assembly into a heteromeric protein complex that consists of two type I and two type II subunits. Within this complex, type II subunits phosphorylate type I subunits at their N-terminal domain. Thus, activated, type I subunits recruit and phosphorylate receptor SMADs (R-smad), which are the first intracellular mediators of TGF-β signalling. TGF-β1 acts predominantly through phosphorylation of the C-terminal MH2 domains of SMAD2 and SMAD3 via activin receptor-like kinase 5 (ALK5), of which activity is located at the C-terminal part of TGFBR1. Upon phosphorylation, R-SMADs recruit the co-SMAD SMAD-4 to form protein complexes that translocate into the nucleus and exhibit transcriptional activity.

In the lung, TGF-β1 has multiple roles in health and disease (summarized in [51]). During embryonic stages of lung organogenesis, TGF-β1 is pivotal in epithelial-mesenchymal interaction, during alveolarization and for airway branching morphology. It is believed that TGF-β1 signalling is involved in controlling epithelial cell proliferation, to drive epithelial-mesenchymal transition (EMT) and it plays a major role in lung fibrosis and emphysema. Furthermore, more recent studies demonstrated that TGF-β1 signalling controls epithelial cell migratory behaviour independent from EMT when activated by irradiation [45] and in conjunction with EMT when activated by TGF-β1 [37].

Lung epithelial cells form a barrier that lines airway and alveolar surfaces and constitute an air-liquid interface. Establishing and maintaining the air-liquid interface needs balanced ion and water transport as well as an appropriate paracellular barrier that ensures diffusion of solvent and solute between airway surface and interstitium while preventing uncontrolled leakage. Tight junctions form the diffusion barrier that seals the paracellular gap between neighbouring epithelial cells. They consist of claudins and occludin. Claudins form an independent protein family. According to their sequence homology, they are subdivided into classic claudins with high sequence homology and non-classic claudins with higher sequence variability [32]. Regardless of the subfamily, all claudins share a common topology with cytoplasmic N- and C-terminal regions, four transmembrane spanning segments and two extracellular loop regions, ECL1 and ECL2. They assemble via cis-interaction to form parallel strands that surround cells at their apico-lateral side [40, 41]. To build up a diffusion barrier that seals the lateral space, claudins of opposing cells interact via their extracellular domains. While ECL2 region confers a holding and narrowing function, the interaction of ECL1 domains mediates the tightness or pores to tight junctions [33]. Hence, the claudin composition of tight junctions determines tight junction morphometric properties [43], conformational dynamics and permeability or functional properties [69]. Claudin expression pattern in lung epithelia differs significantly between segments of the bronchial tree. Claudin 3, 4 and 18 are the most abundantly expressed claudins of the alveolar epithelium [15]. Also, claudin 5 and claudin 7 are expressed in alveolar epithelial cells but only at modest levels. Nonetheless, they are reported to impact tightness of alveolar epithelia [7]. Expression pattern of claudins in epithelial cells along the conductive airways differs significantly from the alveoli. Along these bronchial segments, the epithelium expresses claudin 1, 2, 3, 4, 5, 7 and 8 [10, 26, 27, 30]. Claudins 1, 3, 4 and 8 decrease tight junction permeability and confer charge selectivity to tight junction pores [2, 10, 28, 30] while claudin 5 has an opposing effect on tight junctions of the bronchiolar epithelium and rather increases their permeability [10]. Tight junction formation is the classical or canonical claudin function. However, claudins localize more often rather than not to cellular sides other than tight junctions and non-canonical functions of claudins must be considered. In the airway epithelium, claudin 2 localizes at intracellular sides [27] and the claudins 1, 4 and 7 localize along the basolateral membrane of epithelial cells [10]. Their function at these “non-tight junction sides” still remains rather unknown. They might serve as a claudin pool for tight junction protein recycling [70] or being part of the cellular interface to the extracellular matrix [14, 36]. Also nuclear localization was reported for claudin 1, 2, 3 and 4 especially in tumour cells [11, 24, 34, 65] suggesting that claudins may act as modulators of gene transcription, cell proliferation and migration.

TGF-β1 modulates TJ permeability. Chronic alcohol ingestion triggers TGF-β1 production and release in lung macrophages which disturbs alveolar barrier function [12]. TGF-β1 also exacerbates alveolar barrier damage in endotoxemia [5] and modifies TJ response to granulocytes/macrophage colony stimulating factor (GM-CSF) [47]. Even though these studies address the effects of TGF-β1 on alveolar epithelia, studies on TGF-β1 and eosinophil-driven remodelling in lung tissue suggest that TGF-β1 may also act on epithelia of conductive airways. Herein we addressed the effects of TGF-β1 on TJ of ciliated human bronchiolar epithelia. We demonstrated that TGF-β1 is sensed by motile cilia that are decorated with TGF-β1 receptors 1 and 2 (TGFBR1 and TGFBR2, respectively) and acts on TJ via its canonical SMAD2-dependent signalling pathway. TGF-β1 increases TJ permeability via initiating the shuttling of CLDN3 from the TJ into the nuclei and finally the loss of epithelial integrity. This mechanism might contribute to epithelial damage in inflammatory lung diseases that are driven by eosinophils like atopic asthma.

## Materials and methods

### Cell culture, hBEpC

To coat polyester Transwell-clear filter inserts (3470 0.33 cm^2^ surface area, VWR, Bruchsal, Germany), 100 μl of Collagen I/III solution (Stemcell Technologies, Cologne, Germany) at a dilution of 1: 100 in phosphate-buffered saline solution (Biochrom, Berlin, Germany) was added onto filter inserts and air dried at room temperature. Afterwards, collagen was cross-linked by UV irradiation for 30 min and filters were stored at 4 °C until usage.

Primary human bronchial epithelial cells (hBEpC) were either isolated from fresh tissues that were obtained during tumour resections or lung transplantation with fully consent of patients (Ethics approval: ethics committee medical school Hannover, project no. 2699-2015) or obtained from Epithelix (Geneva, Switzerland). For proliferation, 10^6^ Passage 1 cells were thawed and transferred into cell culture flask (75 cm^2^ surface area, Sarstedt AG & Co. KG, Nümbrecht, Germany) with 20 ml airway epithelial growth medium (Promocell, Heidelberg, Germany) supplemented with 5 μg/ml Plasmocin prophylactic, 100 μg/ml Primocin and 10 μg/ml Fungin (all from InvivoGen, Toulouse, France) and were cultivated at submerged conditions at 37 °C, 5% CO2 and 95% humidity until reaching 90% confluence. Cells were detached using DetachKit (Promocell, Heidelberg, Germany), and 3.5 * 10^4^ cells suspended in 200 μl airway growth medium per filter were transferred to collagen-coated filters placed in 600 μl airway growth medium and cultivated at submerged conditions at 37 °C, 5% CO2 and 95% humidity. Cells reached confluence within 3 to 4 days. Afterwards, air-liquid interface (ALI) conditions were established by aspirating apical medium and basolateral medium was replaced by ALI medium [17] (DMEM-H and LHC basal medium (41966-029, 12677-019, Gibco, Thermo Fisher Scientific, Germany) mixed 1:1 and supplemented with the following: insulin 0.87 μM, hydrocortisone 0.21 μM, epidermal growth factor 0.5 ng/ml, triiodothyronine 0.01 μM, transferrin 0.125 μM, epinephrine 2.5 μM, bovine pituitary extract 10 μg/ml, bovine serum albumin 0.5 mg/ml (all from Promocell, Heidelberg, Germany), phosphorylethanolamine 0.5 μM, ethanolamine 0.5 μM, zinc sulfate 3 μM, retinoic acid 0.05 μM, ferrous sulfate 1.5 nM, calcium chloride 0.6 μM, magnesium chloride 0.11 μM, sodium selenite 30 μM, manganese chloride 1 μM, sodium silicate 0.5 nM, ammonium molybdate tetrahydrate 1 μM, ammonium metavanadate 5 μM, nickel sulfate 1 μM, tin chloride 0.5 μM (all from Sigma-Aldrich/Merck, Darmstadt, Germany), 100 U/ml penicillin and 100 μg/ml streptomycin (both from Gibco, Thermo Fisher Scientific, Germany)). Medium was renewed every other day. From cultivation day 17 onwards mucus was removed from apical surfaces every second day using PBS equilibrated at 37 °C, 5% CO_2_ and 95% humidity. Compounds were added to the basolateral medium at given concentrations and for given time intervals.

### TEER measurement

Transepithelial electrical resistance (TEER) was quantified by using the cellZscope (Nano Analytics, Muenster, Germany). Prior to measurements, the basal electrode was covered with 500 μl pre-heated ALI medium per well. Filters were placed into the wells and 250 μl ALI medium was added to the apical surface. After positioning the apical electrodes, measurements were started immediately. For data acquisition and analysis, the latest software version provided with the cellZscope (NanoAnalytics, Münster, Germany) was used.

### Paracellular permeability

Paracellular permeability was quantified as the apparent permeability coefficient (P_app_) for Na^+^-Fluorescein, 4 kDa or 20 kDa Dextran labelled with fluorescein isothiocyanat (all from Sigma-Aldrich/Merck, Darmstadt, Germany). hBEpC epithelia were placed in 24-well plates containing 600 μl ALI medium with 300 μM of Na^+^-Fluorescein, 4 mg/ml 4 kDa and 20 kDa Dextran, respectively. One hundred ten microlitres of isotonic saline solution (Fresenius Kabi, Bad Homburg, Germany) was added to the apical compartment of hBEpC epithelia and incubated for 1 h at 37 °C, 5% CO_2_ and 95% humidity. Afterwards, two 50-μl aliquots from liquid of the apical compartment were transferred into a 96-well multiwell plate (Nunc, Thermo Fisher Scientific, Germany). Dilution series were prepared for calibration of measurements: for 4 kDa and 20 kDa Dextran (in μg/ml): 0.0, 0.1, 0.5, 1, 2.5, 5, 10, 25, 50 and 100; for Na^+^-Fluorescein (in μM): 0.0, 0.1, 0.5, 1, 2, 4, 5, 6, 8, 10 and 25. Fluorescence intensities were measured using an Infinite M200 plate reader equipped with i-control 2.0 software for data acquisition and analysis (Tecan Group AG, Männerdorf, Switzerland). Slope and *y*-axis intersection of calibration curves were obtained from linear regression calculated through data points of calibration measurements. Concentrations of Na^+^-Fluorescein, 4 kDa and 20 kDa Dextran were determined by measuring fluorescence intensity of apical solution samples according to the following: C = (I_ap_ − c)/m with C as the concentration of the diffusible fluorescent molecule, I_ap_ as the fluorescence intensity measured in the apical solution sample, c as the *y*-axis interception of the calibration curve and m as the slope of the calibration curve. P_app_ was calculated according to the equation: P_app_ = (C * V_0_)/(Δt * A * ΔC_0_) with C as the apical concentration of the fluorescent diffusion marker, V_0_ as the total apical volume at time point 0, Δt as the incubation time (1 h), A as the epithelial surface area (0.33 cm^2^) and ΔC_0_ as the basolateral concentration of the fluorescent diffusion marker at time point 0.

### Semi-quantitative real-time RT-PCR (qRT-PCR)

Total RNA was isolated from cells grown at ALI conditions with or without TGF-β1 1 ng/ml (HZ-1131, Humanzyme via Biomol, Hamburg, Germany). Cell lysis was accomplished by using the cell lysis buffer provided with the mybudget RNA mini kit (Bio-Budget, Krefeld Germany). RNA isolation was performed according to the manufacturer’s protocol with an additional DNase digestion (79254, Qiagen, Hilden, Germany). RNA concentration was quantified via the NanoDrop 2000c spectral photometer (Thermo Fisher Scientific, Germany), and 600 to 800 ng total RNA was used to synthesize cDNA with the SuperScript VILO cDNA Synthesis Kit (Thermo Fisher Scientific, Germany). qRT-PCR was performed using my-Budget 5x EvaGreen (R) QPCR-Mix II (Bio-Budget, Krefeld Germany) and Quantitect primer assays (Table 1, all from Qiagen, Hilden, Germany). The RealPlex2 thermocycler provided with latest version of RealPlex data acquisition and analysis software (Eppendorf, Hamburg, Germany) was used. PCR program was as follows: initial denaturation and Taq activation at 95 °C for 15 min followed by 40 cycles with denaturation at 95 °C for 15 s, primer annealing and amplification at 60 °C for 20 s and a final extension at 72 °C for 20 s. For PCR product verification, melting curves were determined by a final denaturation at 95 °C for 15 s, annealing at 60 °C for 15 s and heat ramping from 60 °C to 95 °C with a ramp duration of 20 min. Crossing points (C_t_) were defined using the CalQPlex method provided with the thermocycler data acquisition and analysis software. Gene expression was determined as duplets, and relative expression levels were calculated according to the following: E_rel_ = 2^ΔCt^ with E_rel_ as expression level relative to expression levels of calibrator gene and ΔC_t_ as: ΔC_t_ = C_t(gene of interest)_−C_t(calibrator)_ with ΔC_t_ as the threshold cycle difference, C_t(gene of interest)_ as the threshold cycle of gene of interest and C_t(calibrator)_ as the threshold cycle of housekeeping gene used as calibrator.

**Table 1:** Quantitect primer assays used in semi-quantitative real-time RT-PCR (all from Qiagen, Hilden, Germany)

Gene (human)	Primer assay	Gene (human)	Primer assay
POLR2A	QT00033264	CLDN12	QT01012186
CLDN1	QT00225764	CLDN14	QT00234731
CLDN2	QT00089481	CLDN15	QT00202048
CLDN3	QT00201376	CLDN16	QT00039655
CLDN4	QT00241073	CLDN17	QT00209923
CLDN5	QT00232197	CLDN18	QT00039550
CLDN6	QT00235193	CLDN19	QT00083475
CLDN7	QT00236061	CLDN20	QT00218057
CLDN8	QT00212268	TJP1	QT00077308
CLDN9	QT00209482	TGFBR1	QT00083412
CLDN10	QT00031101	TGFBR2	QT00014350
CLDN11	QT00008085	TGFBR3	QT00083223

### Immunocytochemistry

hBEpC were washed for three times with phosphate-buffered saline (PBS) without Ca^2+^ and Mg^2+^ (Biochrome GmbH, Berlin, Germany) and subsequently fixed with 2% (*w*/*v*) paraformaldehyde (Sigma-Aldrich/Merck, Darmstadt, Germany) in PBS for 10 min at room temperature (RT). After aspiration, remaining fixation buffer was removed by repetitive washing with PBS followed by incubation for 10 min at room temperature in 1 M glycine in PBS (Sigma-Aldrich/Merck, Darmstadt, Germany). Afterwards, cells were incubated in ice-cold methanol/acetone (1:1) mixture for 2 min at room temperature followed by three repetitive washing steps with PBS. Cell membrane permeabilization was performed by incubation in PBS supplemented with 0.5% Triton X-100 (Sigma-Aldrich/Merck, Darmstadt, Germany) for 10 min at room temperature followed by blocking step by incubation with PBS + 0.5% Triton X-100 + 2% fetal bovine serum (FBS) (Sigma-Aldrich/Merck, Darmstadt, Germany) for 10 min at room temperature. All primary antibodies (Table 2) were diluted in PBS + 2% FBS, and cells were incubated in antibody dilution for 1 h at room temperature. Unbound antibodies were removed by subsequent washing three times with PBS + 2% FBS. Bound antibodies were detected by incubation with secondary antibody dilution (all 1: 400 in PBS + 2% FBS). As secondary antibodies, the following were used: Alexa Fluor-488 donkey anti-rabbit IgG, Alexa Fluor-568 donkey anti-goat IgG, Alexa Fluor-647 donkey anti-mouse IgG, Alexa Flour-488 donkey anti-mouse IgG (all from Invitrogen, Germany), Alexa Fluor-405 donkey anti-rat IgG (ab175670, Abcam, Cambridge, UK). Cell nuclei were counterstained with Hoechst 33342 (Invitrogen, Germany). Again, unbound antibodies were removed by subsequent washing steps with PBS + 2% FBS. hBEpC filters were mounted on microscope slides (Superfrost Plus, Thermo Fisher Scientific, Germany) using the ProLong™ Diamond Antifade Mountant (Thermo Fisher Scientific, Germany). Image acquisition was performed by using a Leica TCS SP5 confocal microscope with HCX PL APO CS 40 × 1.25 oil, HCX PL APO lambda blue 63 × 1.4 oil objectives and corresponding software Leica Application suite (Leica, Wetzlar/Mannheim Germany). Images for the blue (Hoechst 3342), green (AlexaFluor 488), red (AlexaFluor 568) and far-red (AlexaFluor 647) channels were taken in sequential mode using appropriate excitation and emission settings.

**Table 2:** Used antibodies

Antigen	Antibody	Vendor	Annotation
CLDN1	anti-claudin 1, rabbit polyclonal	Abcam plc., Cambridge, UK	Cat. No.: ab15098, RRID: AB_301644, dilution 1:500
CLDN3	anti-claudin 3, rabbit polyclonal	Abcam plc., Cambridge, UK	Cat. No.: ab15102 RRID: AB_301648 dilution 1:100
CLDN4	anti-claudin 4, rabbit polyclonal	Abcam plc., Cambridge, UK	Cat. No.: ab53156 RRID: AB_869176 dilution: 1:500
CDH1	anti-E-cadherin, rabbit polyclonal	Abcam plc., Cambridge, UK	Cat. No.: ab15148 RRID: AB_301693 dilution: 1:500
TUBG1	anti-gamma tubulin 1, mouse, monoclonal	Abcam plc., Cambridge, UK	Cat. No.: ab11316 RRID: AB_297920 dilution: 1:500
TGFBR1	anti-TGF beta Receptor I, rabbit, polycolonal	Abcam plc., Cambridge, UK	Cat. No.: ab31013 RRID: AB_778352 dilution: 1:500
TGFBR2	anti-TGF beta Receptor II [MM0056-4F14], mouse monoclonal	Abcam plc., Cambridge, UK	Cat. No.: ab78419 RRID: AB_1603198 dilution: 1:100
TGFBR3	anti-TGF beta Receptor III/TGFBR3 antibody [MM0057-5G9], mouse monoclonal	Abcam plc., Cambridge, UK	Cat. No.: ab78421 RRID: AB_2202598 dilution: 1:100
SMAD2 – phosphorylated	anti-phospho-SMAD2 (Ser465, Ser467), rabbit polyclonal	Thermo Fisher Scientific, Germany	Cat. No.: 44-244G RRID: AB_2533614 dilution: 1:100
SMAD2	anti-SMAD2, mouse monoclonal (5G7)	Thermo Fisher Scientific, Germany	Cat. No.: MA5–15877 RRID: AB_11153038 dilution: 1:500
SMAD2	anti-SMAD2, Rabbit monoclonal [EP784Y]	Abcam plc., Cambridge, UK	Cat. No.: ab40855 RRID:AB_777977 dilution: 1:250
TUBA1A	anti- alpha Tubulin, rat monoclonal (YL1/2)	Thermo Fisher Scientific, Germany	Cat. No.: MA1–80017 RRID: AB_2210201 dilution: 1:1000
HSP90AA1/AB1	anti-HSP90alpha/beta, mouse monoclonal (F-8)	Santa Cruz, Heidelberg, Germany	Cat. No.: sc-13119 RRID: AB_675659 dilution: 1:500
anti-Rabbit IgG	IRDye® 680RD Donkey anti-Rabbit IgG (H + L)	Licor Biotechnologies GmbH, Bad Homburg, Germany	Cat. No.: 926-68073 RRID: AB_10954442 dilution: 1:20,000
anti-Mouse IgG	IRDye® 800CW Donkey anti-mouse IgG (H + L)	Licor Biotechnologies GmbH, Bad Homburg, Germany	Cat. No.: 926-32212, RRID: AB_621847 dilution: 1:20,000

Colocalization Analysis was performed to quantify the amount of pSMAD2-C and CLDN3 at basal bodies of motile cilia, at TJs and in the nucleus using the IMARIS 9.3 software (Bitplane AG, Zurich, Switzerland). In brief, Voxel background intensity was estimated using Gaussian filter and estimated background was subtracted. Afterwards, 3D pseudo-surfaces of regions of interests (ROI) were calculated as volumes that are positive for Hoechst 33342 as Cell nuclei, positive for γ-tubulin (γTUB) as basal bodies or positive for zonula occludens 1 (ZO-1) as TJs. Colocalization of γTUB, ZO-1 and Hoechst 33342 with pSMAD2-C or CLDN3 was analysed according to the method of Costes and Lockett [9]. Enrichment of pSMAD2-C at basal bodies, TJs and inside the nucleus was measured by quantifying voxels within ROI at which colocalization was detected.

Tight junction organization ratio (TiJOR) was measured using latest version of ImageJ according to Terryn and colleagues [64]. In brief, TJ structures were identified from confocal images after immune staining of hBEpC for ZO-1 and CLDN3. To convert grey scale images into binary images, the latest version of ImageJ software and the ridge detection plugin [57, 72] was used and the number of intersections of TJ stricter to a mask of concentrically arranged rectangles with increasing perimeter was determined. The TiJOR was calculated as the mean of numbers of intersections normalized to rectangle perimeter and is given as intersections per μm (ints./μm).

### Immunohistochemistry

Slices (1.5 to 3 μm) from formalin-fixed and paraffin-embedded airway tissue biopsies were obtained from the tissue library of the Institute for Pathology (MHH, Medical School Hannover). Slices were deparaffinized via incubating slices in Xylol (Carl Roth, Karlsruhe, Germany) for 2 × 10 min at RT, followed by incubation steps in a descending alcohol series for 10 min. Antigen retrieval and antigen detection were performed by using the ZytoChem Plus (HRP) One-Step Polymer anti-mouse/rabbit/rat, together with the DAB Substrate Kit (Zytomed Systems, Berlin, Germany) according to manufacturer’s protocol. TGFBR1 and TGFBR2 were detected with primary antibodies given in Table 2. After mounting on microscope slides (Superfrost Plus, Thermo Fisher Scientific, Germany) using the Eukitt® Quick-hardening mounting medium (Sigma-Aldrich/Merck, Darmstadt, Germany), tissue slices were assessed microscopically using a Leica DM6000B microscope with HC PLAN APO 20x/0.70 PH 2, HC PLAN APO 40x/0.75 PH 2 objectives and corresponding software Leica Application Suite X (Leica, Wetzlar/Mannheim Germany). All experiments were performed with approval of the ethics committee of the Hannover Medical School (Project no. 2699-2015, approved April 2015).

### Western blotting

For lysis of hBEpC, cells were incubated for 10 min on ice in 30 μl protein lysis buffer with 50 mM TRIS-HCl pH 7.2, 1 mM EDTA, 1 mM EGTA and 1% Triton X-100 (all from Sigma-Aldrich/Merck, Darmstadt, Germany) supplemented with cOmplete™ Mini Protease Inhibitor Cocktail and PhosSTOP™ (both Roche, Basel, Switzerland). Afterwards, 10 μl 4× protein loading buffer (LI-COR Biotechnologies GmbH, Bad Homburg, Germany) supplemented with NuPAGE reducing agent (Thermo Fisher Scientific, Germany) was added to cell lysates. Protein denaturation was performed at 70 °C for 10 min. Proteins were separated on Bolt™ 4–12% Bis-Tris Plus polyacrylamide gels using a mini gel tank system containing NuPAGE™ MOPS SDS running buffer supplemented with NuPAGE antioxidant (all from Thermo Fisher Scientific, Germany). The Chameleon Duo Pre-stained protein ladder (LI-COR Biotechnologies GmbH, Bad Homburg, Germany) was used as size marker. After gel electrophoresis, proteins were transferred to nitro cellulose membranes using the iBlot Gel transfer system together with the iBlot nitrocellulose transfer stacks (both from Thermo Fisher Scientific, Germany) according to manufacturer’s protocol. Protein transfer was verified by Ponceau S staining (Sigma-Aldrich/Merck, Darmstadt, Germany). Afterwards, membranes were washed in Tris-buffered saline (TBS) (Merck Millipore, Darmstadt, Germany) and subsequently incubated in Odyssey TBS blocking buffer (LI-COR Biotechnologies GmbH, Bad Homburg, Germany) containing 50 mM sodium fluoride (Sigma-Aldrich/Merck, Darmstadt, Germany).

Proteins were detected using anti-claudin 3 and anti-HSP90 a/b primary antibodies (see Table 2), diluted in an Odyssey blocking buffer containing 0.2% TWEEN 20 and 50 mM sodium fluoride (both from Sigma-Aldrich/Merck, Darmstadt, Germany). Claudin-3 and HSP90 a/b antibody incubation was performed over night at 4 °C. Unbound antibodies were removed by subsequent washing steps with TBS containing 0.2% TWEEN 20 (TBS-T) followed by incubation in Odyssey blocking buffer containing 0.2% TWEEN 20, 50 mM sodium fluoride and secondary antibodies with a 1:20,000 dilution for 1 h at RT. Secondary antibodies used: IRDye® 680RD Donkey anti-Rabbit IgG (H + L) and IRDye® 800CW Donkey anti-Mouse IgG (H + L) (both from LI-COR Biotechnologies, Bad Homburg Germany). Non-bound antibodies were removed by washing membranes in TBS-T and TBS as final washing step. Proteins were detected with the LI-COR Odyssey Fc Near Infrared Fluorescence Detection System with corresponding image acquisition and analysis software (LI-COR Biotechnologies, Bad Homburg Germany).

### Statistic

Prism 6 (GraphPad, San Diego, USA) was used for data analysis. Statistical tests used are given in the text. Differences were considered statistical significant when *p* < 0.05, and significance levels were depicted in the figures as follows: *p < 0.05, **p < 0.01, ***p < 0.001.

## Results

### TGF-β1 increases paracellular permeability

**Fig. 1:**
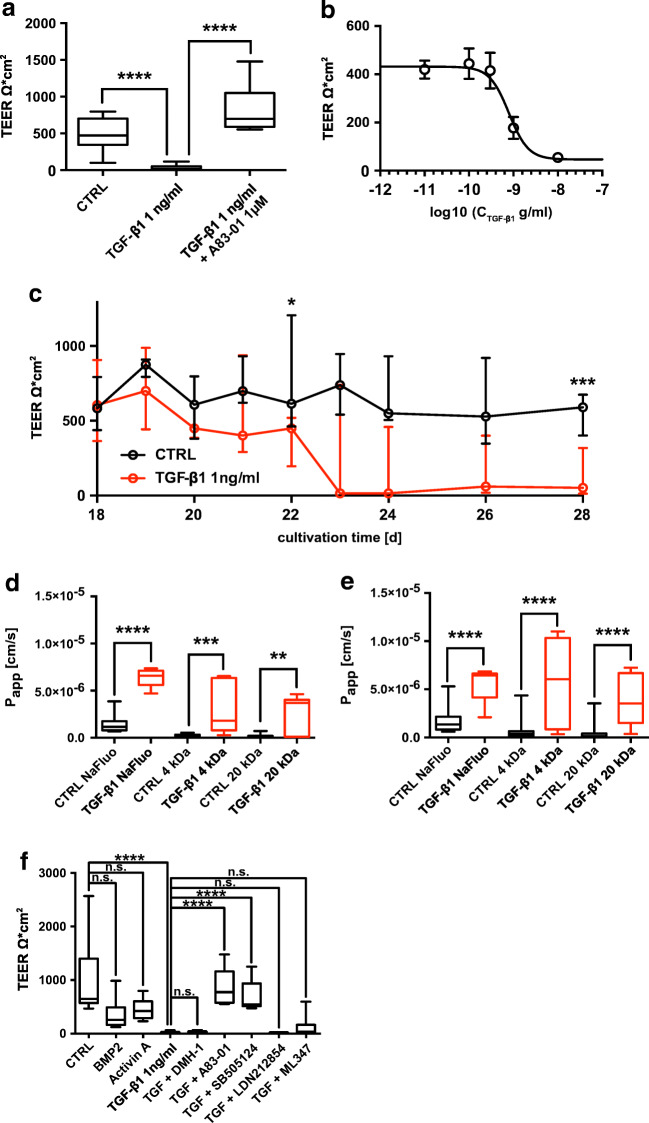
Response of hBEpC epithelia to TGF-β1. hBEpC were cultivated at air-liquid interface conditions until day 18. TGF-β1 was added to the basolateral medium on day 18 (TGF-β1). Control cells (CTRL) remained untreated. **a** TEER was measured on cultivation day 28 when treated cells were exposed to 1 ng/ml TGF-β1 or TGF-β1 + 1 μM A83-01 for 10 days. TGF-β1 reduced TEER when compared to control cells. A83-01 abolished TGF-β1-induced TEER reduction **b** Concentration response of hBEpC cells to TGF-β1. TGF-β1 was added at given concentrations. Data points represent mean TEER ± SEM (*N* = 11 to 12 from 3 donors). Concentration response curve was calculated as best fit through data points according to hill-equation with a hill-slope of 2 (see text). **c** Time course of hBEpC epithelia to 1 ng/ml TGF-β1. TGF-β1 induces a decrease in TEER from day 20 onwards. The drop in TEER was significant on day 22 (Mann-Whitney test *p* = 0.0122 CTRL vs TGF- β1) and also at the endpoint of time course on day 28 (Mann-Whitney test *p* = 0.0006 CTRL vs TGF-β1). Data points represent median and error bars inter-quartile range (all *N* = 8 to 9 from 3 independent donors. Values are given in supplementary information Table S1). Apparent permeability coefficient of hBEpC control epithelia and TGF-β1-treated epithelia to sodium-fluorescein (Na-fluo), 4 kDa and 20 kDa dextran (4 kDa and 20 kDa, respectively). **d** Measured on day 22 after 4 days of TGF-β1 exposure. All comparisons: CTRL versus TGF-β1, Mann-Whitney test, epithelia were obtained from cells of 3 donors, *N* = 8–9. Values are given in supplementary information Table S2. **e** Measured on day 28 after 10 days of TGF-β1 exposure. All comparisons: CTRL versus TGF-β1, Mann-Whitney test, epithelia were obtained from cells of 3 donors. Box plots represent median as line, boxes quartile and whiskers minimum to maximum ranges. Values are given in supplementary information Table S2. **f** TGF-β1 but neither bone morphogenic protein BMP2 (2 ng/ml) nor Activin A (100 ng/ml) reduce TEER. ALK inhibitors A83-01 and SB505124 abolished TGF-β1-induced TEER reduction

In the present study, we cultivated hBEpC at ALI conditions for 18 days in order to maintain a differentiated epithelium with sustained ALI, a transepithelial electrical resistance (TEER) > 300 Ωcm^2^, mucus secretion and ciliated cells with beating cilia. Therefore, it reflected almost all properties of bronchial epithelia. To test whether TGF-β1 has any effect on epithelial tightness and barrier function (Fig. 1a and Table S1 supplementary material), we quantified the transepithelial electrical resistance (TEER) after exposing cells to TGF-β1 (1 ng/ml added to the basolateral medium) for 10 days before assessing TEER on day 28 of ALI culture (Fig. 1a). TGF-ß1 treatment significantly reduced TEER; this effect was abolished by ALK5 inhibitor A83-01 (final concentration 1 μM). The TGF-β1 effect was concentration dependent (Fig. 1b) with an IC_50_ = 0.8 ng/ml (95% confidence interval, CI ranging from 0.42 ng/ml to 1.5 ng/ml), TEER_min_ = 46.5 Ωcm^2^ (CI = 0 Ωcm^2^ to 146.1 Ωcm^2^) and TEER_max_ = 431.6 Ωcm^2^ (CI = 382.5 Ωcm^2^ to 480.7 Ωcm^2^). Time course experiments revealed that TGF-β1 does not cause an immediate TEER reduction (Fig. 1c). Between day 19 and 20, we observed a drop in TEER in control epithelia and TGF-β1-treated epithelia. However, this drop appeared slightly more intense in TGF-β1-treated epithelia. On day 22, the reduction of TEER in TGF-β1-treated epithelia was significant when compared to control epithelia. Between day 22 and 23, TGF-β1 TEER further declined in TGF-β1-treated epithelia while no change in TEER was observed in control epithelia. The drop in TEER was accompanied by an increase in apparent permeability coefficient (P_app_) for the small diffusible molecules sodium-fluorescein (Na-fluo) and 4 kDa dextran as well as for the large diffusible molecule 20 kDa dextran. The increase in P_app_ for the tested molecules was evident on cultivation day 22 after 4 days of TGF-β1 exposure (Fig. 1d and Table S2, supplementary material) and on cultivation day 28 after 10 days of TGF-β1 exposure (Fig. 1e and Table S2, supplementary material). Hence, the reduction of TEER in TGF-β1-treated epithelia was due to an increase in paracellular permeability. To further elaborate whether TGF-β1 acts on paracellular permeability through ALK5 or possibly through alternative pathways, we tested activin A and bone morphogenic protein BMP2 as two other TGF-related ligands on TEER. Furthermore, we tested different ALK inhibitors for their effect on TGF-β1-induced TEER reduction (Fig. 1f and Table S3 supplementary material). BMP2 and Activin A both reduced the TEER but to a much lower extent than TGF-β1 did. Inhibitors that lack any potency to inhibit ALK5 (DMH-1, ALK2 inhibitor; LDN212854, ALK1 and 2 inhibitor and ML347, ALK1 and 2 inhibitor) did not abolish the TGF-β1 effect on TEER. A83-01 (ALK 4, 5 and 7 inhibitor) and SB505124 (ALK4 and 5 inhibitor) both abolished TGF-β1-induced drop in TEER. They are similar in blocking ALK5 but differ in their efficiency to inhibit other ALKs. Taken together, these experiments demonstrate that TGF-β1 acts through TGFR1 that harbours ALK5 activity.

### TGF-β1 receptors localize on motile cilia

**Fig. 2:**
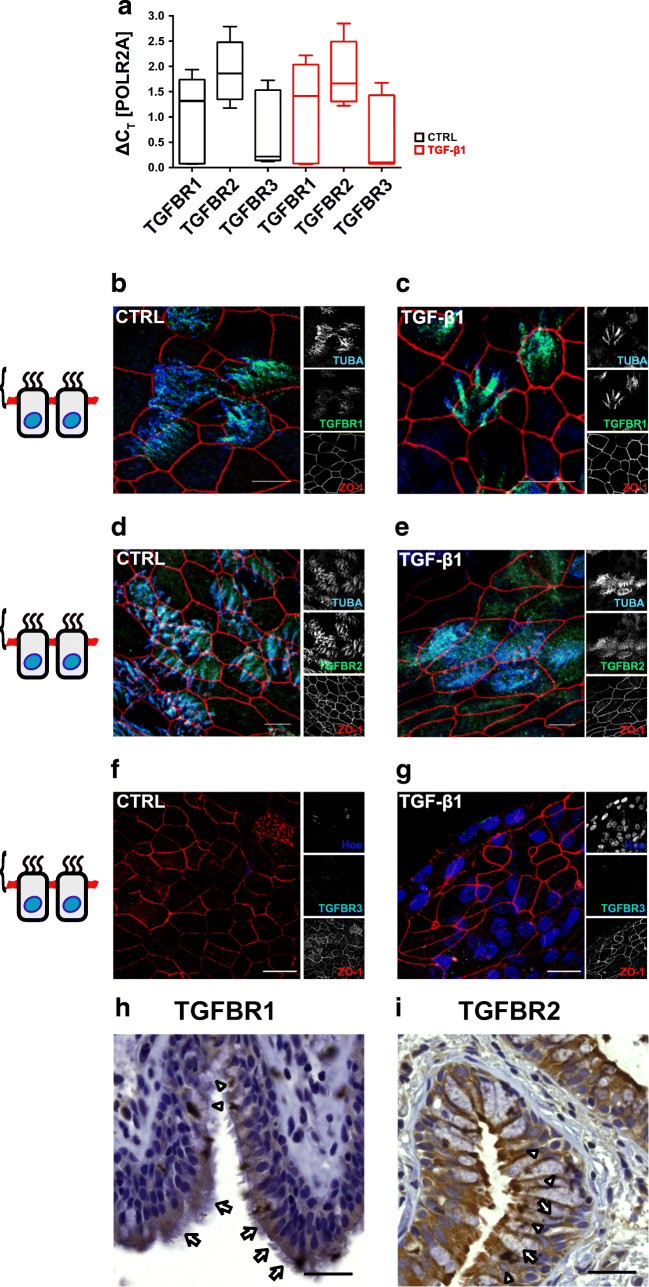
**a** TGF-β1 receptors TGFR1, -R2, and -R3 expression relative to RNA polymerase II (POL2RA) levels. Data given as median, boxes represent quartile ranges and whiskers minimum to maximum ranges (all *N* = 12 from three different donors). **b**–**g** TGF-β1 receptors type 1 (TGFBR1) and type 2 (TGFBR2) localize on motile cilia whereas TGFR3 was not detected in immunocytochemical stainings. Z-stacks of confocal images (brackets in the schemes at the left hand side depict focus levels of Z-stacks) of primary cultivated human bronchial epithelial cells. Control cells remained untreated (CTRL) and TGF-β1 cells were exposed for 10 days from cultivation day 18 to cultivation day 28 (TGF-β1). RGB colour images represent merged grey scale images given with the small insets at the right. α-tubulin (TUBA, blue channel) were used as counterstaining for motile cilia, zonula occludens 1 (ZO-1, red channel) was used as counterstaining for tight junctions and TGFBR1, TGFBR2 and TGFR3 (green channel) gives localization of TGF-β1 receptor isoforms. Scale bar 10 μm. Immunostaining of paraffin-embedded lung slices for **e** TGFBR1 and **f** TGFBR2. Ciliated cells (highlighted by arrows) are positive for both receptor isoforms. Non-ciliated cells (highlighted by triangles) showed almost no expression for either receptor isoform. Scale bars 30 μm

Three TGF-β1 receptor subunits are known so far, TGFR1, -R2 and -R3. While TGFR1 and -R2 dimerization initiates ALK5 activation [22, 63], TGFR3 acts as a co-receptor that may facilitate ligand binding to TGFR1 and -R2 subunits [54]. To elucidate expression levels of all three receptor subunits, we performed RT-PCR experiments (Fig. 2a). These experiments revealed highest expression levels for TGFR1 and -R2, while TGFR3 expression was by magnitudes lower, even though the expression levels varied between different donors. To identify cellular localization of TGF-receptors, we performed immunofluorescence labelling experiments for all three receptors (Fig. 2b–g). In these experiments, we used ZO-1 and alpha-tubulin (TUBA) as a counterstaining for TJ and motile cilia, respectively. In control epithelia, TGFBR1 and TGFBR2 localize specifically to motile cilia and were almost absent in non-ciliated epithelial cells (Fig. 2b and d). While TGFBR1 localization was not affected by TGF-β1 (Fig. 2b, c), TGFBR2 localization changed upon TGF-β1 treatment. In control epithelia, TGFBR2 was selectively expressed on motile cilia (Fig. 2d), whereas in cells exposed to TGF-β1, TGFBR2 was found on motile cilia as well as in cellular compartments of epithelial cells that are not positive for TUBA, which we used as a marker for motile cilia (Fig. 2e). Immunostaining experiments failed to detect any TGFR3 protein in either control and TGF-β1-treated cells (Fig. 2f, g). Immunohistochemical experiments on human lung tissue revealed similar expression patterns for both receptors (Fig. 2h, i) in airway epithelia. TGFBR1 and -R2 were again detected in ciliated but not in non-ciliated epithelial cells.

### TGF-β1 acts via SMAD2-dependent signalling

**Fig. 3:**
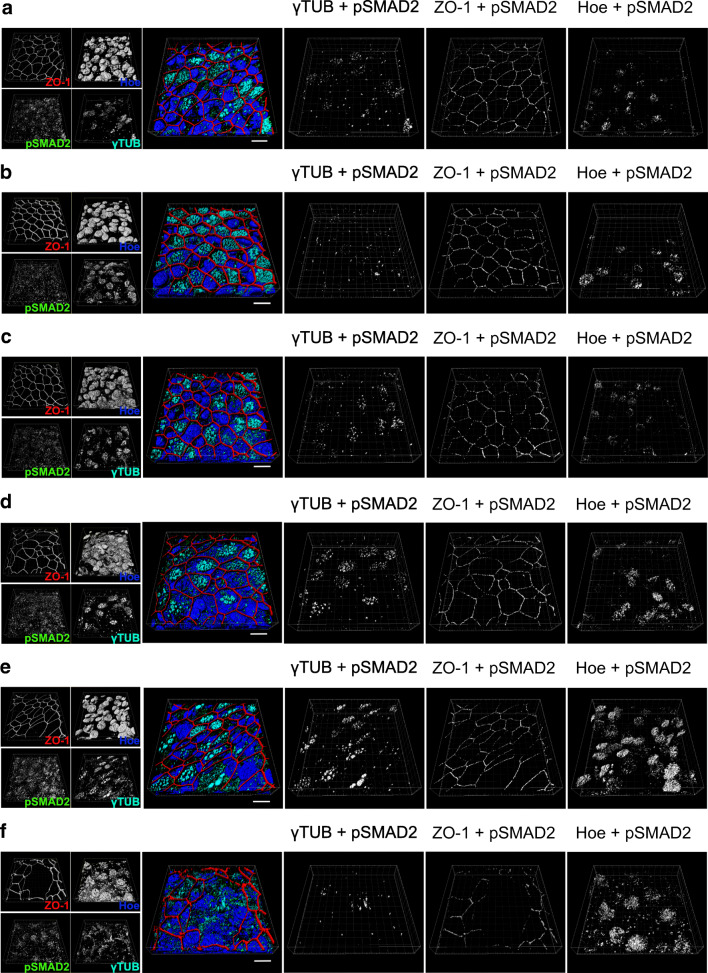
TGF-β1 initiates accumulation of pSMAD2-C (pSMAD2) at centrioles of motile cilia and cell nuclei. **a**–**c** hBEpC cultivated at ALI control conditions (CTRL) for 19, 22 and 28 days. **d**–**e** hBEpC were cultivated at ALI conditions and exposed to TGF-β1 from cultivation day 18 to 28 (TGF-β1). Colour images give 3D pseudosurfaces for zonula occludens 1 (ZO-1, red), cell nuclei counterstained by Hoe33342 (Hoe, blue), pSMAD2-C (pSMAD2, green) and γ-tubulin as centriole counterstaining (γTUB, cyan). Scale bar 10 μm, small insets at the right give single channel grey scale images. Large grey scale images to the right represent 3D pseudosurfaces of overlapping volumes for γ-tubulin and pSMAD2-C (γTUB + pSMAD2), ZO-1 and pSMAD2-C (ZO-1 + pSMAD2) and nuclear regions with overlapping pSMAD2-C staining (Hoe + pSMAD2) **a** CTRL d19 **b**. CTRL d22 **c**. CTRL d28 **d**. TGF-β1 d19 **e**. TGF-β1 d22 **f**. TGF-β1 d 28

**Fig. 4:**
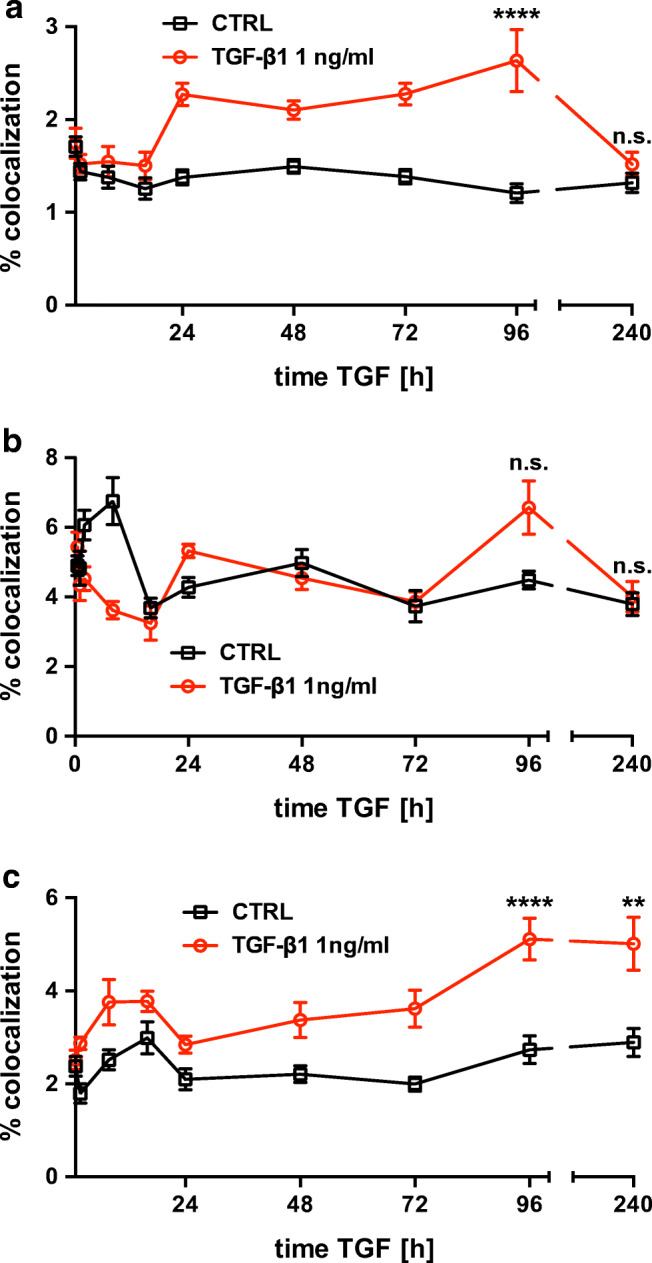
Time course of pSMAD2-C (pSMAD2) in hBEpC cultivated at ALI control conditions (CTRL) and hBEpC cultivated at ALI in the presence of TGF-β1 from cultivation day 18 to 28. Data gives volumes positive for pSMAD2-C in % of **a** γ-tubulin positive volume (pSMAD2 + γTUB), TGF-β1 versus control at time points, 96 h *p* < 0.0001 and 240 h *p* = 0.63; b zonula occludens 1 positive volume (pSMAD2 + ZO-1), TGF- β1 versus control at time points, 96 h *p* = 0.24 and 240 h *p* > 0.99; and **c** Hoechst 3342 positive volume (pSMAD2 + Hoe), TGF-β1 versus control at time points, 96 h *p* < 0.0001 and 240 h *p* = 0.0046. Data points summarize results from analysis of images given in Fig. 3 as mean ± SEM (*N* = 18 to 21, from 4 donors). All statistics tests: Kruskal-Wallis test with Dunn’s correction for multiple comparisons **a**) pSMAD2 + γTUB **b**) pSMAD2 + ZO-1 **c**) pSMAD2 + Hoe

Our observation that A83-01 abolished TGF-β1-induced TEER reduction provides strong evidence that TGF-β1 acts via ALK5-dependent signalling pathways. The effector molecule of ALK5 is the receptor activated phosphorylated SMAD2 [20]. Hence, we investigated intracellular localization of phosphorylated SMAD2 (pSMAD2-C) in time course experiments (Fig. 3, Fig. S1 and Fig. S2). In these experiments, ALI cultivated epithelia were exposed to TGF-β1 (1 ng/ml) from day 18 of ALI culture onwards. Control cells remained untreated for the same time interval. pSMAD2-C co-localized with tight junction protein zonula occludens 1 (ZO-1), Hoechst-33342 (nucleus) and the basal body of motile cilia (y-TUB) in control epithelia (Fig. 3a–c and Fig. S1), indicating basal pSMAD2-C levels even under control conditions. However, TGF-β1 exposure caused an increase of pSMAD2-C at the basal bodies within 1 day (cultivation day 19) of TGF-β1 exposure and remained elevated for at least 72 h (day 22) before returning to basal levels after 10 days (day 28) (Fig. 3d–e, Fig. S2 and Fig. 4a). In contrast, pSMAD2 protein density did not change at the tight junctions during 10 days of TGF-β1 treatment (Fig. 3d–e, Fig. S2 and Fig. 4b). Upon receptor-mediated phosphorylation, pSMAD2 forms heteromers with SMAD3 and SMAD4 that results in its translocation into the nucleus. This is considered an indication for on-going activation of TGF-β1/SMAD2 signalling. Indeed, we observed persistent pSMAD2 accumulation in nuclei after 48 h of TGF-β1 treatment (day 20) (Fig. 3d–e, Fig. S2 and Fig. 4c). Surprisingly, even though both TGFBR1 and -R2 receptors are solely expressed in ciliated epithelial cells and TGF-β1 treatment resulted in pSMAD2-C accumulation at the basal bodies as the earliest measured response, pSMAD2-C were also enriched in nuclei of non-ciliated cells.

**Fig. 5:**
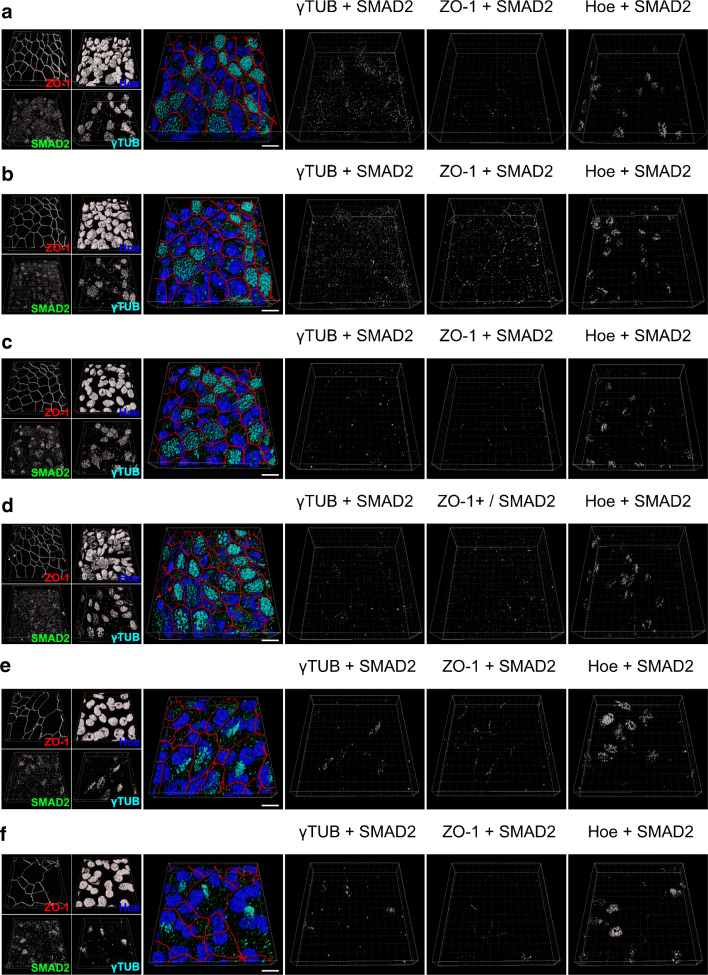
Effect of TGF-β1 on intracellular localization of non-phosphorylated SMAD2. **a–c** hBEpC cultivated at ALI control conditions (CTRL) for 19, 22 and 28 days. **d**–**e** hBEpC were cultivated at ALI conditions and exposed to TGF-β1 from cultivation day 18 to 28 (TGF-β1). Colour images give 3D pseudosurfaces for zonula occludens 1 (ZO-1, red), cell nuclei counterstained by Hoechst 33342 (Hoe, blue), SMAD2 (SMAD2, green) and γ-tubulin as centriole counterstaining (γTUB, cyan). Scale bar 10 μm, small insets at the left give single channel grey scale images. Large grey scale images to the right represent 3D pseudosurfaces of overlapping volumes for γ-tubulin and SMAD2 (γTUB + SMAD2), ZO-1 and SMAD2 (ZO-1+ SMAD2) and nuclear regions with overlapping SMAD2 staining (Hoe + SMAD2) **a** CTRL d19 **b**. CTRL d22 **c**. CTRL d28 **d**. TGF-β1 d19 **e**. TGF-β1 d22 **f**. TGF-β1 d28

**Fig. 6:**
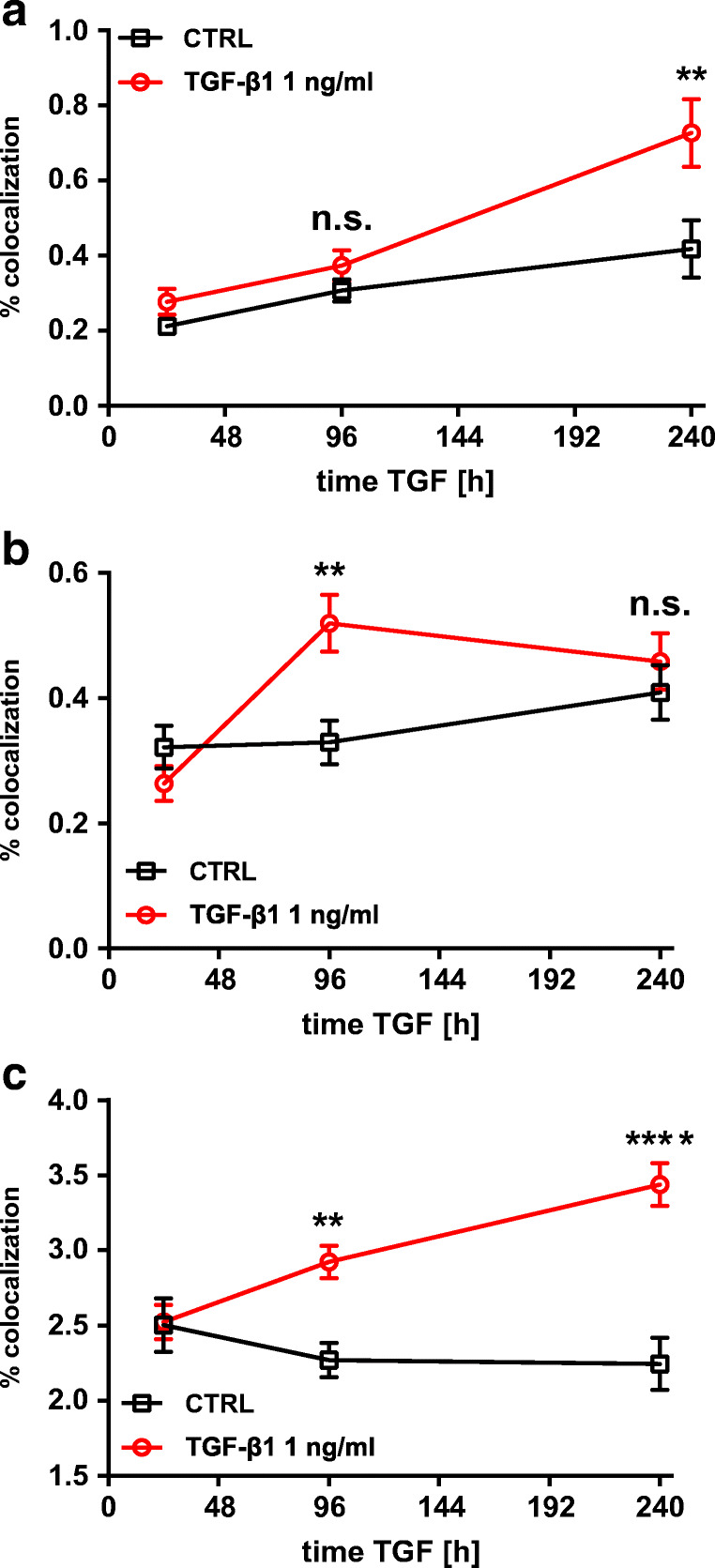
Time course of SMAD2 (SMAD2) in hBEpC cultivated at ALI control conditions (CTRL) and hBEpC cultivated at ALI in the presence of TGF-β1 from cultivation day 18 to 28. Data gives volumes positive for SMAD2 in % of **a** γ-tubulin positive volume (SMAD2 + γTUB), TGF-β1-treated cells show a significantly higher colocalization compared to untreated control cells on day 28 (time point 240 h of TGF-β1 exposure) but not on day 22 (time point 96 h of TGF-β1 exposure), TGF-β1 versus control time point 96 h *p* = 0.56 and 240 h *p* = 0.0038. **b** Zonula occludens 1 positive volume (SMAD2 + ZO-1), TGF-β1-treated cells show a significantly higher colocalization compared to untreated cells on day 22 but not on day 28 (96 h and 240 h of TGF-β1 treatment, respectively), TGF-β1 versus control time points 96 h *p* = 0.0087 and 240 h *p* = 0.81, and **c** Hoe positive volume (SMAD2 + Hoe), TGF-β1-treated cells show a significantly higher colocalization compared to untreated cells on day 22 and day 28 (96 h and 240 h of TGF-β1 treatment respectively) TGF-β1 versus control for time point 96 h *p* = 0.0047 and 240 h *p* < 0.0001. Data points summarize results from analysis of images given in Fig. 3 as mean ± SEM (*N* = 18 to 21, from 4 donors). All statistic tests: Kruskal-Wallis with Dunn’s correction for multiple comparisons **a**. SMAD2 + γTUB **b**. SMAD2 + ZO-1 **c**. SMAD2 + Hoe

We further tested whether TGF-β1 treatment affected intracellular localization of non-phosphorylated SMAD2. In control epithelia (Fig. 5a–c, Fig. 6 and Fig. S3), non-phosphorylated SMAD2 was observed in all cells and a minor proportion co-localized with y-TUB at the basal body of motile cilia, with ZO-1 at the TJ and was also evident in cell nuclei. TGF-β1 treatment affected this intracellular localization slightly but significantly. In TGF-β1-treated cells (Fig. 5d–f, Fig. 6 and Fig. S4), an elevated localization of non-phosphorylated SMAD2 at the basal body of motile cilia became evident on cultivation day 28 (after 10 days of TGF-β1 exposure, Fig. 6a). Its localization at the TJ was also elevated on cultivation day 22 (after 4 days of TGF-β1 exposure, Fig. 6b) and non-phosphorylated SMAD2 localization also increased over time upon TGF-β1 treatment (Fig. 6c). However, the proportion of voxels being positive for SMAD2 colocalization with y-TUB, ZO-1 and with nuclear fluorescence marker Hoechst-33342 was by a magnitude smaller when compared with the colocalization of p-SMAD2-C in control and TGF-β1-treated epithelia. No differences in SMAD2 protein abundance were observed in TGF-β1-treated versus control epithelia, neither on cultivation day 19 nor on cultivation day 28 (supplementary information Fig. S5).

### TGF-β1 triggers redistribution of CLDN3

**Fig. 7:**
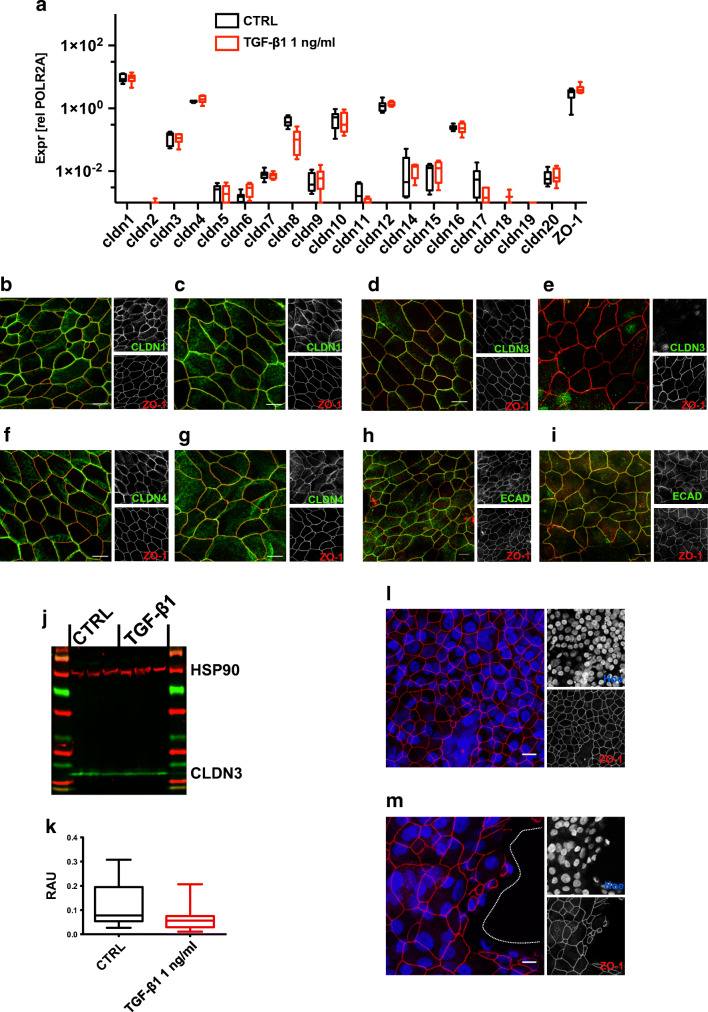
Effect of TGF-β1 exposure on Claudin composition of tight junctions and lateral contacts. **a** Claudin expression levels measured by semi-quantitative RT-PCR experiments on hBEpC cultivated at ALI control conditions (CTRL) and after 10 days of TGF-β1 exposure (TGF-β1) on cultivation day 28. Expression levels are given as expression relative to POLR2A and were summarized as median; boxes represent quartile range and whiskers minimum to maximum range (all *N* = 9 to 12, from 4 donors). Representative confocal images of immunostainings on hBEpC cultivated at ALI control conditions for 22 cultivation days (CTRL) and after 4-day TGF-β1 exposure on cultivation day 22 (TGF-β1) for **b**, **c** claudin 1 (CLDN1, green); **d**, **e** claudin 3 (CLDN3, green); **f**, **g)** claudin 4 (CLDN4, green); and **h**, **i** for E-cadherin (ECAD, green). Zonula occludens staining (ZO-1, red) was used as tight junction counterstaining. RGB images represent merged channels and small insets at the right are single channel grey scale images. Scale bar 10 μm. **j**, **k** Western blot experiments demonstrate no significant difference in CLDN3 protein abundance in hBEpC cultivated at ALI control conditions (CTRL) at cultivation day 22 versus hBEpC exposed to TGF-β1 for 4 days from cultivation day 18 to 22 (TGF-β1). Heat shock protein HSP90 was quantified as internal calibrator. Box plot gives protein abundance of CLDN3 normalized to HSP90 abundance as relative arbitrary units (RAU). Data summarized as median, boxes represent inter-quartile range and whiskers minimum/maximum range (*N* = 9, from 3 donors). **l** Z-projection of confocal image demonstrates that epithelial integrity remained intact in hBEpC on cultivation day 28. **m** Z-projection of confocal images shows that epithelial integrity was disturbed in hBEpC exposed to TGF-β1 for 10 days from cultivation day 18 to 28. Larger regions with epithelial lesions could be observed (dashed line depicts interface between areas with lesion and with contacting cells). Hoechs33342 (Hoe, blue) was used as nuclear and zonula occludens 1 (ZO-1, red) tight junction staining. Scale bars 10 μm **b**. CTRL **c**. TGF-β1 **d**. CTRL **e**. TGF-β1 **f**. CTRL **g**. TGF-β1 **h**. CTRL **i**. TGF-β1 **l**. CTRL **m**. TGF-β1

TJs limit paracellular diffusion and their functional properties depend on their claudin (CLDN) composition [31]. To identify possible TGF-β1-induced changes in CLDN expression levels, we performed semi-quantitative real-time RT-PCR experiments (Fig. 7a). However, none of the tested CLDN genes showed a significant change in expression level upon TGF-β1 treatment of hBEpC epithelia. To test whether the TGF-β1-induced TEER decrease depends on possible alterations in sub-cellular CLDN distribution, we compared intracellular claudin distribution in control versus TGF-β1-treated epithelia. As mentioned above, we observed that TEER was significantly reduced on cultivation day 22 (after 4 days of TGF-β1 exposure) in TGF-β1-treated vs. non-treated control cells. It also was the last time point prior to the final drop in TEER that occurred between cultivation day 22 and 23 in TGF-β1-treated epithelia. Therefore, we investigated protein localization at the TJ after 4 days of TGF- β1 exposure on cultivation day 22. We focused on CLDN1 (Fig. 7b, c), CLDN3 (Fig. 7d, e) and CLDN4 (Fig. 7f, g). These claudins were the most abundantly expressed claudins in the herein investigated epithelia that are also referred to confer sealing properties to the TJs in airway epithelia [52]. In addition, we investigated subcellular localization of E-cadherin (ECAD, Fig. 7h, i) to evaluate lateral contacts between neighbouring cells. No difference in TJ localization in CTRL versus TGF-β1-treated cells could be observed for CLDN1 and CLDN4. In addition, no difference was observed for ECAD localization in CTRL versus TGF-β1-treated cells. In contrast, CLDN3 disappeared from the TJ within 4 days of exposure to TGF-β1 (Fig. 7d, e). In all experiments, counterstaining of ZO-1 contoured epithelial cells at their apico-lateral side and was not affected by TGF-β1. Thus, 4 days of TGF-β1 treatment did not affect lateral contact of neighbouring epithelial cells as well as the structural integrity of TJ. However, the absence of CLDN3 at TJ in TGF-β1-treated cells was striking. Since CLDN3 confers tightening properties to TJ [10], our observation could explain the increase in paracellular permeability as well as the drop in TEER. Western blot experiments revealed no change in CLDN3 protein abundance in CTRL versus TGF-β1-treated cells (Fig. 7j, k). Evidently, loss of CLDN3 protein at the TJ is not due to a reduction in cellular CLDN3 protein levels. Since we observed a massive increase in paracellular permeability after cultivation day 22 (compare Fig. 1), we performed complementing TJ staining on day 10 of TGF-β1 exposure (Fig. 7l, m). These experiments revealed areas with ZO-1 staining pattern that contoured epithelial cells at their apico-lateral side in both, control and TGF-β1-treated epithelia. However, regions of sporadic isolated cells interspersed regions with almost intact confluent cell layers in TGF-β1-treated epithelia. Thus, dissociation of epithelial cells and formation of larger lesions accompanied this drop of TGF-β1-induced epithelial damage.

**Fig. 8:**
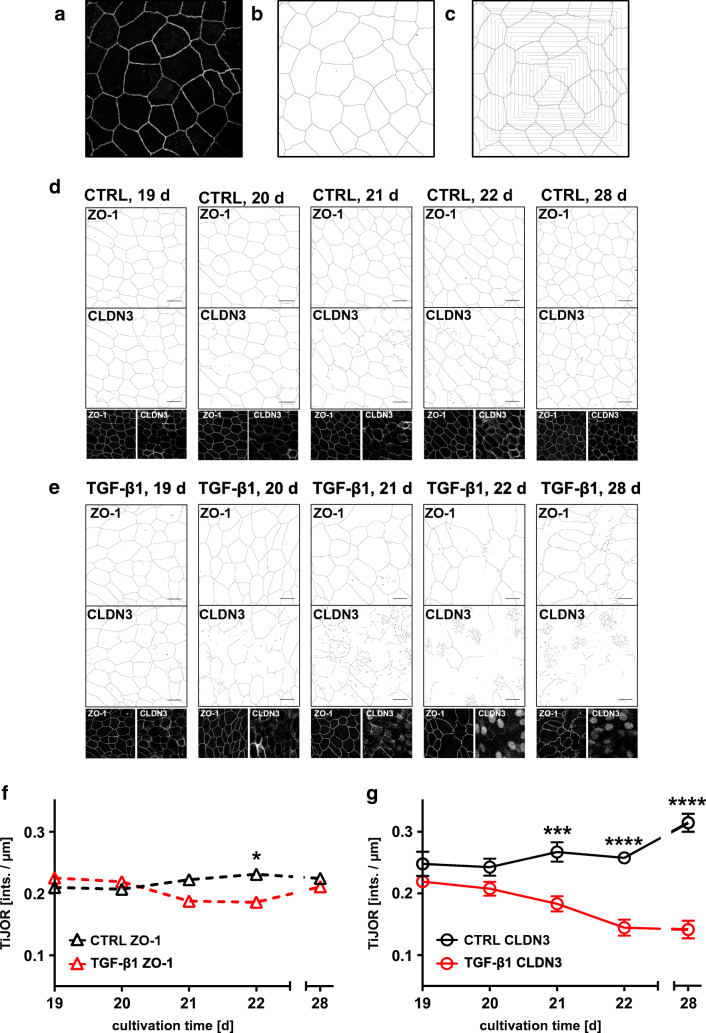
Analysis of tight junction (TJ) integrity using the tight junction organization ratio (TiJOR). To demonstrate analysis procedure, **a** confocal grey scale images of hBEpC immune stained for TJ proteins were used for TJ detection. **b** TJs were identified and images were converted into binary images using the Ridge Detection tool of ImageJ. **c** Number of intersections of TJ structures to a mask of concentric rectangles was determined. TiJOR is calculated as number of intersections (ints.) normalized to perimeter of rectangles in ints./μm. **d** TiJOR image analysis of confocal images of hBEpC cultivated at ALI control conditions at cultivation day 19, 20, 21, 22 and 28, and **e** of hBEpC exposed to TGF-β1 for 1 day, 2 days, 3 days, 4 days and 10 days (cultivation time 19 days, 20 days, 21 days, 22 days, 28 days, respectively). Cells were immune stained for zonula occludens 1 (ZO-1) as TJ counterstaining and claudin 3 (CLDN3). Binary images represent detected TJ structures as described above for ZO-1 and CLDN3 signals. Small insets below give grey scale images. **f**, **g** Time course for TiJOR summarizes image analysis for ZO-1 (**f**) and CLDN3 (**g**) immune staining of hBEpC control and TGF-β1-treated epithelia. Data are summarized as mean ± SEM (*N* = 18, from 3 donors). TiJOR tends to be elevated for ZO-1 staining in control versus TGF-β1-treated epithelia, of which difference became significant on day 22 only (*p* = 0.0226). However, TiJOR for CLDN3 staining was elevated in control versus TGF-β1-treated epithelia from day 21 onwards (day 21 *p* = 0.0002, day 22 *p* < 0.0001 and day 28 *p* < 0.0001). All statistic tests: Kruskal-Wallis test with Dunn’s correction for multiple comparisons

**Fig. 9:**
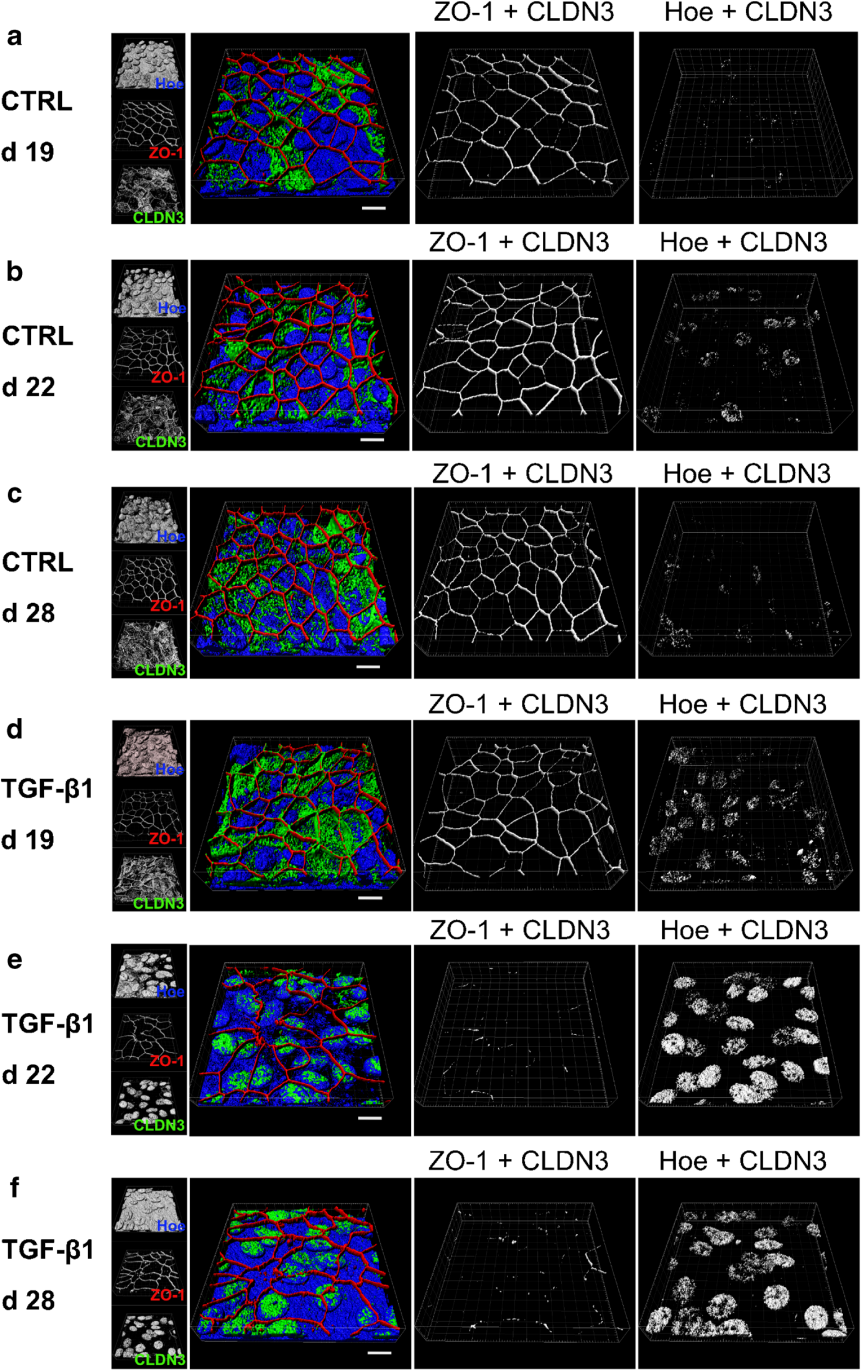
TGF-β1 initiates redistribution of claudin 3 from tight junctions into cell nuclei. hBEpC epithelia were cultivated at **a**–**c** ALI control conditions (CTRL) for 19 days, 22 days and 28 days and exposed to **d**–**f** TGF-β1 from cultivation day 18 to days 19, 22, and 28 (corresponding exposure times: 1 day, 4 days and 10 days, respectively). RGB images represent merged 3D pseudosurface images for claudin 3 (CLDN3, green), zonula occludens 1 as TJ counterstaining (ZO-1, red) and Hoechst 33342 as nuclear counterstaining (Hoe, blue). Scale bar 10 μm. Single channel 3D pseudosurface images are given with the small insets at the left. Grey scale images represent 3D pseudosurface images of volumes that are positive for ZO-1 and CLDN3 (ZO-1 + CLDN3) as well as for Hoe and CLDN3 (Hoe + CLDN3)

**Fig. 10:**
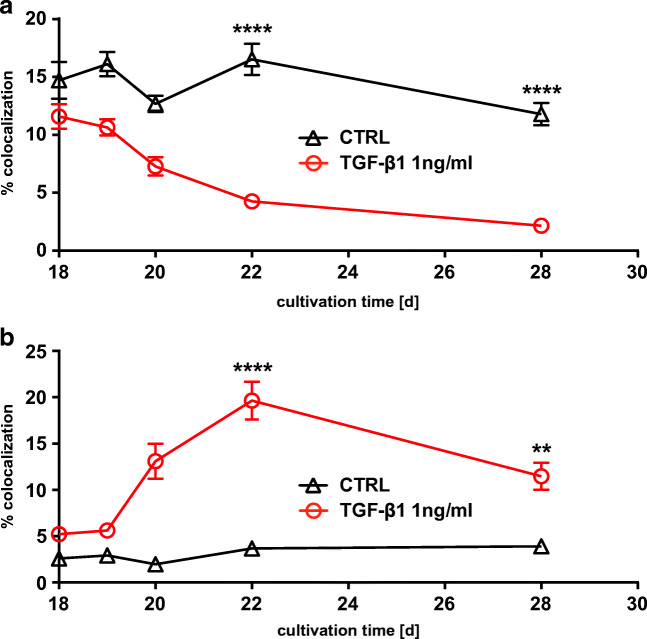
Time course of change in intra-cellular CLDN3 localization. **a** Change of CLDN3 at tight junctions quantified as volume being positive for CLDN3 and ZO-1 normalized to volume being positive for ZO-1 in %. TGF-β1 treatment reduced CLDN3 colocalization with ZO-1. TGF-β1 versus control at time points day 22 *p* < 0.0001 and day 28 *p* < 0.0001). **b** Change in CLDN3 in cellular nuclei quantified volume being positive for CLDN3 and Hoechst-33342 (Hoe) normalized to volumes being positive for Hoe in %. TGF-β1 treatment increases CLDN3 in cell nuclei. TGF-β1 versus control at time points day 22 *p* < 0.0001 and day 28 *p* = 0.0025). Data points represent mean ± SEM (*N* = 18 from 3 donors). All statistic tests: Kruskal-Wallis test with Dunn’s correction for multiple comparisons **a**. CLDN3 + ZO-1 **b**. CLDN3 + Hoe

To elucidate the fate of CLDN3 in TGF-β1-treated cells, we investigated its subcellular localization over time. First, we investigated the CLDN3 organization at the TJ by measuring tight junction organization ratio (TiJOR) according to the protocol of Terryn and colleagues [64]. This method quantifies the discontinuity in protein distribution along TJs as TiJOR rather than measuring protein abundance at the TJs. We identified TJ structures from immune staining for ZO-1 and CLDN3 on hBEpC epithelia [57, 72] (Fig. 8a, b) and calculated TiJOR as intersections of TJ with concentrically arranged rectangles of increasing perimeter length (Fig. 8c). In control cells, the immune staining for ZO-1 and CLDN3 both revealed an almost continuous staining of TJ structures that contour the cells (Fig. 8d). The TJ staining in TGF-β1-treated cells (Fig. 8e) revealed that ZO-1 remained contouring the cells even on cultivation day 28 that corresponded to 10 days of TGF-β1 exposure. However, TJ morphology changed and spikes that protrude almost perpendicularly from the TJ were observed. TiJOR analysis of ZO-1 staining deviated on day 21 and 22 in TGF-β1-treated cells from control cells (Fig. 8f). The observed difference was significant on day 22 only (control vs TGF-β1-treated cells, Kruska-Wallis test with Dunn’s correction for multiple comparison *p* = 0.023, *N* = 18 each). Since ZO-1 staining pattern was contouring cells almost without any interruptions, this deviation may reflect changes in cell shape upon TGF-β1 exposure. However, on day 28, no difference between control and TGF-β1-treated cells could be observed. Contrary to that, the staining pattern of CLDN3 became patchy and discontinuous. This was also evident from the TiJOR analysis of CLDN3 staining pattern (Fig. 8g). In control cells TiJOR of CLDN3 staining slightly increases over time most likely due to changes in cell shape as a result of prolonged cultivation time. TiJOR significantly dropped from day 21 onwards in TGF-β1-treated versus control cells. This drop indicates the loss of continuity of CLDN3 staining during TGF- β1 exposure and reflects the fact that CLDN3 did not localize at the TJ. The loss of CLDN3 proteins at the TJ could explain the reduction in TEER. To elucidate the fate of CLDN3 during TGF-β1 exposure, we performed additional time course experiments to follow the change of the intracellular localization of CLDN3 (Fig. 9). In these experiments, cells were treated with TGF-β1 from cultivation day 18 onwards while control cells remained untreated and cells were immune stained for CLDN3. Staining for ZO-1 and Hoechst-33342 staining were performed as counterstaining for TJ and nuclei, respectively. In control cells, CLDN3 localized predominantly at the TJ and only a smaller proportion was detected in the nuclei (Fig. 9a–c). When cells were exposed to TGF-β1, CLDN3 localization changed remarkably (Fig. 9d–e). At early time points of TGF-β1 treatment, CLDN3 localized predominantly at the TJ, as it is shown in Fig. 9d for cultivation day 19 that corresponds to 1 day of TGF-β1 exposure. On day 4 as well as on day 10 of TGF-β1 exposure, CLDN3 disappeared almost completely from the TJ and only small TJ segments were positive for CLDN3 (Fig. 9e and f, respectively). Analysing the time course of these experiments revealed that removal of CLDN3 from TJs (Fig. 10a) and CLDN3 accumulation in cell nuclei (Fig. 10b) is synchronized. It started after 1 day of TGF-β1 treatment and both were completed 3 days later on day 22. Nuclear CLDN3 accumulation was detected not solely in ciliated but also in non-ciliated epithelial cells. No difference in time course of CLDN3 redistribution from TJs into cell nuclei could be observed in ciliated and non-ciliated cells.

**Fig. 11:**
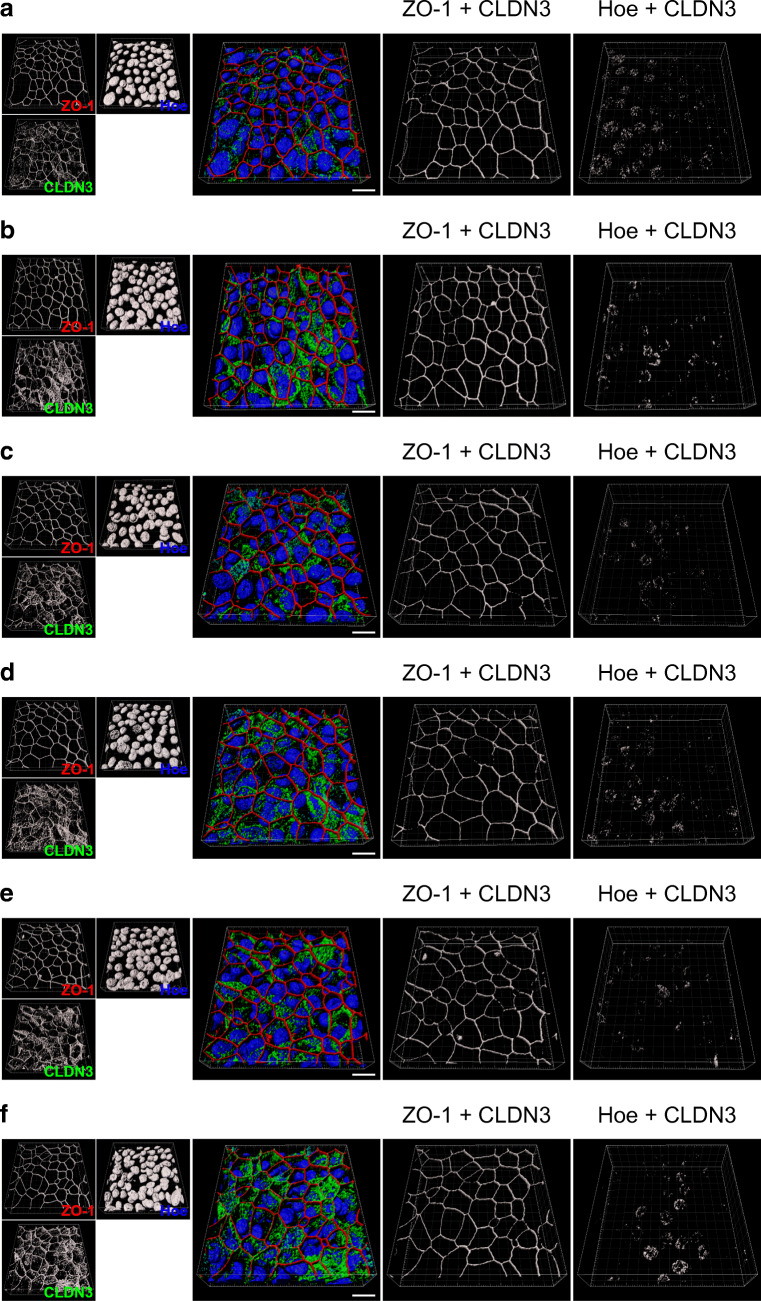
Effect of Activin A receptor type II-like kinase 5 (ALK5) inhibitor A83-01 on intracellular CLDN3 localization. hBEpC epithelia were cultivated at **a**–**c** ALI control conditions (CTRL) for 19 days, 22 days and 28 days and exposed to **d**–**f** the ALK5 inhibitor A8301 from cultivation day 18 to days 19, 22, and 28. RGB images represent merged 3D pseudosurface images for claudin 3 (CLDN3, green), zonula occludens 1 as TJ counterstaining (ZO-1, red), Hoechst 33342 as nuclear counterstaining (Hoe, blue) and SMAD2 (SMAD2, cyan) to analyse possible colocalization between SMAD2 and CLDN3. Scale bar 10 μm. Single channel grey scale images are given with the small insets at the left. Grey scale images represent 3D pseudosurface images of volumes that are positive for ZO-1 and CLDN3 (ZO-1 + CLDN3) as well as for Hoe and CLDN3 (Hoe + CLDN3). Scale bars represent 10 μm **a**. CTRL d19 **b**. CTRL d22 **c**. CTRL d28 **d**. CTRL + A83-01 d19 **e**. CTRL + A83-01 d22 **f**. CTRL + A83-01 d28

**Fig. 12:**
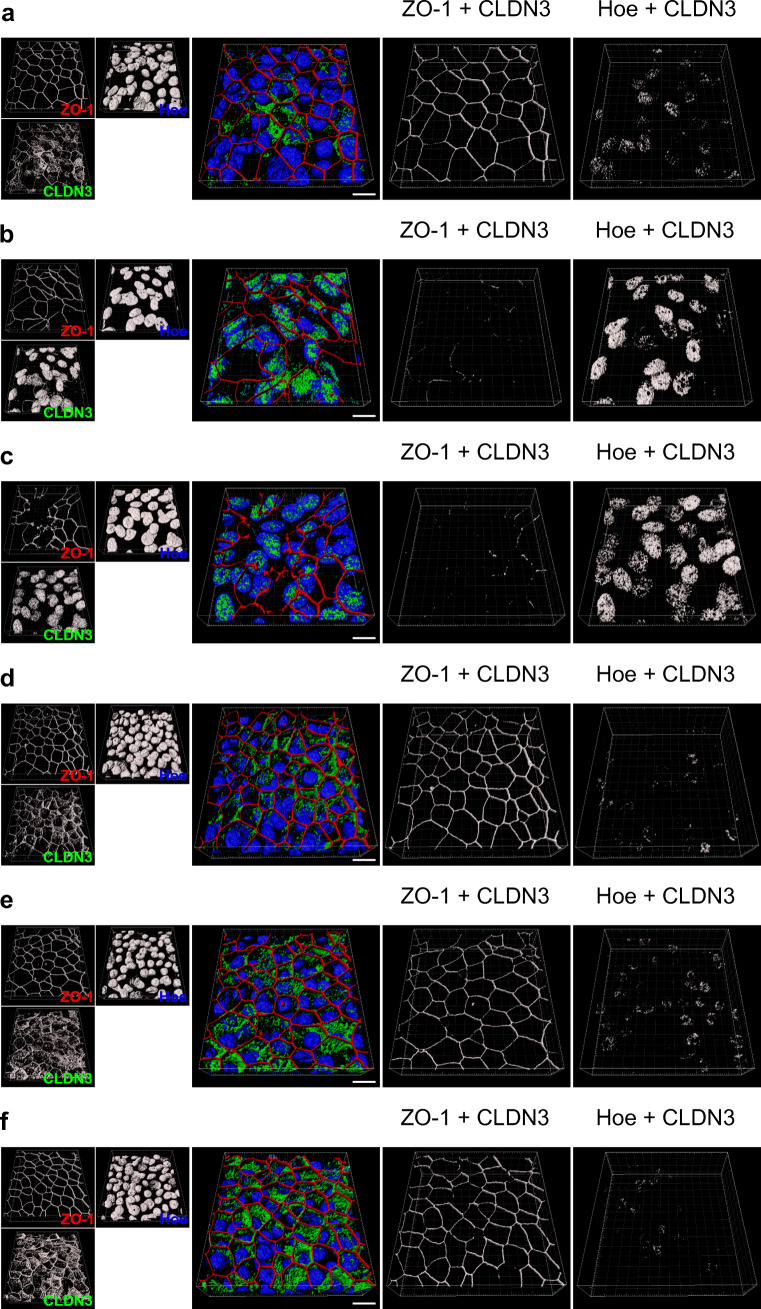
TGF-β1-induced redistribution of claudin 3 from tight junctions into cell nuclei is prevented by activin A receptor type II-like kinase 5 (ALK5) inhibitor A83-01. hBEpC epithelia were cultivated with **a**–**c** TGF-β1 from cultivation day 18 to days 19, 22, and 28 (corresponding exposure times: 1 day, 4 days and 10 days, respectively) and exposed to **d**–**f** TGF-β1 together with the ALK5 inhibitor A8301 from cultivation day 18 to days 19, 22, and 28 (corresponding exposure times: 1 day, 4 days and 10 days, respectively). RGB images represent merged 3D pseudosurface images for claudin 3 (CLDN3, green), zonula occludens 1 as TJ counterstaining (ZO-1, red), Hoechst 33342 as nuclear counterstaining (Hoe, blue) and SMAD2 (SMAD2, cyan) to analyse possible colocalization between SMAD2 and CLDN3. Scale bar 10 μm. Single channel grey scale images are given with the small insets at the left. Grey scale images represent 3D pseudosurface images of volumes that are positive for ZO-1 and CLDN3 (ZO-1 + CLDN3) as well as for Hoe and CLDN3 (Hoe + CLDN3). Scale bars represent 10 μm **a**. TGF-β1 d19 **b**. TGF-β1 d22 **c**. TGF-β1 d28  **d**. TGF-β1 + A83-01 d19 **e**. TGF-β1 + A83-01 d22 **f**. TGF-β1 + A83-01 d28

**Fig. 13:**
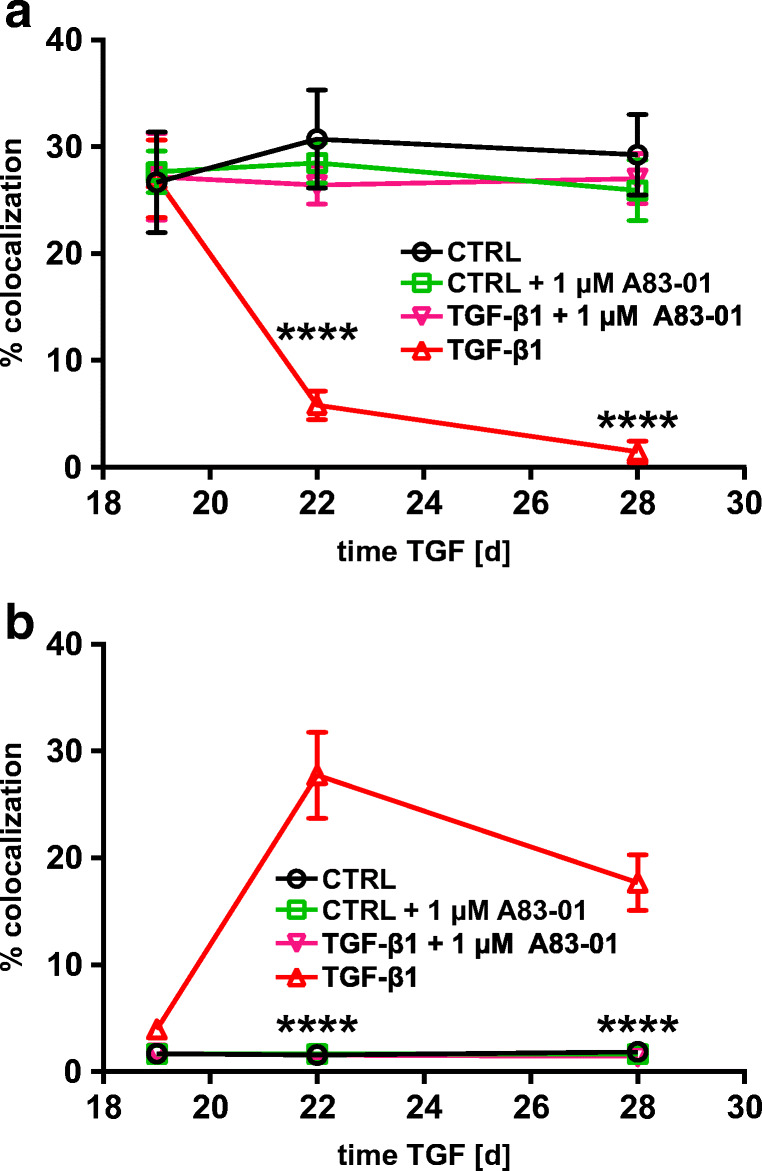
Time course of change in intra-cellular CLDN3 localization. **a** Change of CLDN3 at tight junctions quantified as volume being positive for CLDN3 and ZO-1 normalized to volume being positive for ZO-1 in %. When compared with control cells, TGF-β1 treatment reduced CLDN3 colocalization with ZO-1. This effect was abolished by ALK5 inhibitor A83-01 (TGF-β1 vs. TGF-β1 + A83-01 day 22 *p* < 0.0001 and day 28 *p* < 0.0001, all *N* = 18 from 2 donors, *T* test with Holm-Sidak correction for multiple comparison). **b** Change of CLDN3 abundance in cell’s nuclei quantified as volume being positive for CLDN3 and Hoechst-33342 (Hoe) normalized to volumes being positive for Hoe in %. TGF-β1-induced nuclear CLDN3 accumulation. The ALK5 inhibitor A83-01 abolishes TGF-β1-induced accumulation of CLDN3 in nuclei (TGF-β1 vs. TGF-β1 + A83-01 day 22 *p* < 0.0001 and day 28 *p* < 0.0001, all *N* = 18 from 2 donors, *T* test with Holm-Sidak correction for multiple comparison) **a**. CLDN3 + ZO-1 **b**. CLDN3 + Hoe

To test whether CLDN3 redistribution is initiated by ALK5 activity that depends on TGF-β1 binding to TGFR1/R2 receptors and that triggers SMAD2 phosphorylation, we tested A83-01 as an ALK5 inhibitor on CLDN3 redistribution. A83-01 showed no effect on intracellular CLDN3 localization in control cells (Figs. 11 and 13). However, when added to TGF-β1-treated epithelia, A83-01 abolished CLDN3 redistribution from TJ into cell nuclei almost completely (Figs. 12 and 13). These results make it evident that TGF-β1 initiates the shift of CLDN3 from TJ and its nuclear accumulation.

## Discussion

The lung epithelium separates the luminal compartment of the airways and alveoli from the interstitium. It forms a barrier, which is needed to establish and to maintain an air liquid interface (ALI) that is typical for the lung. It controls the exchange of solvent (water) and solutes between the separated compartments by active transcellular transport processes and by limiting the paracellular diffusion–driven flux of molecules. TJs are sealing the lateral intercellular space of adjacent epithelial cells at their apical side, and thereby they are forming the limiting barrier for paracellular diffusion. Their functional properties depend on their protein composition, especially on their claudin composition [31].

TGF-β1 plays multiple roles in the lung [51], and from recent studies, evidence arose that TGF-β1 directly affects the functional properties of TJs [47, 49, 66, 74]. Herein we addressed the effect of TGF-β1 on the TJ of human lung bronchial epithelial cells and demonstrate that TGF-β1 causes an increase in paracellular permeability. This is not an immediate effect but the drop in TEER started after 1 day of TGF- β1 exposure and was accompanied by the deprivation of CLDN3 from the TJs, while TJ integrity remained almost intact. The formation of destructive lesions within the epithelial cell layer was observed from day 5 of TGF-β1 treatment onwards. Disrupted TJs were observed only in cells that surrounded lesions while TJ remained almost intact in cells within almost undisturbed epithelial areas. TGF-β1 is believed to trigger epithelial-mesenchymal transition (EMT), a gradual process in which epithelial cells acquire mesenchymal phenotype [29]. The loss of contacts between neighbouring epithelial cells that is accompanied with a downregulation and/or redistribution of ZO-1 and E-CAD is accounted as a key step during EMT [76]. We did not observe any redistribution or reduced expression of ZO-1 or E-CAD that preceded lesion formation, and hence, our experiments did not reveal evidence for EMT to cause or to precede epithelial lesion formation.

TGF-β1 is known to activate its receptors TGFBR1 and -R2. Their dimerization upon ligand binding activates ALK5 [10, 20] and subsequently induces the phosphorylation of receptor SMADs, SMAD2 and SMAD3 at serine residues within their C-terminal region [1, 56, 77]. The C-terminal phosphorylated receptor SMADs—p-SMAD2-C and pSMAD3-C—form complexes with SMAD4 and translocate into the nucleus [44]. Our experiments revealed that TGF-β1 induces pSMAD2-C accumulation in the nucleus. Furthermore, we observed that only A83-01 and SB505124 abolished TGF-β1-induced increase in paracellular permeability. Both compounds share the ability to inhibit ALK5 but deviate in their ability to block other ALKs. Inhibitors, which are ineffective to block ALK5 activity failed to impede TGF-β1 effects in airway epithelial cells. These observations show that TGF-β1 acts via its canonical SMAD2/3 pathway to initiate its effect on TJs.

We demonstrated that TGFBR1 and -R2 localize along the shaft of motile cilia of primary cultivated human bronchial epithelial cells as well as of the bronchi of human lung tissue. Motile cilia were recently reported as cellular compartments that have sensory capacity. Several receptor proteins are expressed in the motile cilia of oviduct ciliated cells, like TIE1 and TIE2 [60], the polycystins PKD1, PKD2 and TRPV4 [61] as well as the progesterone receptors PR-A and PR-B [62]. Therefore, it is believed that oviduct motile cilia sense changes in extracellular luminal compartment that occur e.g. during ovulation. Also, motile cilia of the respiratory tract contain multiple receptors like taste receptor isoforms [19, 35, 53, 75], components of hedgehog signalling [38] and the cytokine receptor CX3CR1 [25]. They are believed to be involved in sensing of bacterial factors and noxae in the apical surface liquid layer [19, 35, 38, 53, 75] but can also serve as viral receptors that confer susceptibility to virus infections [25]. Our study adds TGF-β1 receptors as additional receptors to the subset of receptors that localize at upper airways motile cilia. Whereas a role of TGF-β1 receptors for motile cilia sensory capacity is sparsely elaborated, TGF-β1 signalling has been linked to primary cilia. The ciliary pocket at the base of primary cilia serves as a hub for clathrin-mediated endocytosis of TGFBR1/-R2 that facilitates R-SMAD phosphorylation and the accumulation of pSMAD2-C and pSMAD3-C at the basal body of primary cilia [8]. Our observation that pSMAD2-C accumulates at the basal body of motile cilia upon TGF-β1 stimulation points to a mechanism similar to that reported for primary cilia. Activation of TGFR1 triggers its affinity to SMAD2 that serves as ALK5 substrate [23]. Indeed, we observed that non-phosphorylated SMAD2 localized at basal bodies of motile cilia, which was also elevated after 10 days of TGF-β1 exposure, even though non-phosphorylated SMAD2 quantity at basal bodies was rather marginal when compared to pSMAD2-C. Probably SMAD2 phosphorylation upon its binding to TGFR1 subunits is faster than dissociation of pSMAD2-C from the subunit or at least from the side of its phosphorylation. However, it demonstrates that like primary cilia, motile cilia serve as a sensory compartment of TGF-β1 signalling. It further strengthens that TGF-β1 acts via its canonical signalling pathway.

Our experiments revealed that pSMAD2-C also localizes at the TJ even under control conditions. Also, non-phosphorylated SMAD2 was apparent at the TJ. Again, SMAD2 abundance at the TJ was much lower when compared to pSMAD2-C. Evidently, C-terminal phosphorylation facilitates SMAD2 localization at TJ complexes. TGF-β1 exposure did not affect TJ localization neither of pSMAD2-C nor of non-phosphorylated SMAD2. Phosphorylation of SMAD2 at its C-terminal region is mediated by ALK5. It initiates translocation of R-SMAD2, -3/SMAD4 complexes into the nucleus and occurs within its C-terminal region at Ser465 and Ser467 [1, 56]. However, pSMAD2-C that localizes at the TJ seems to escape from nuclear shuttling. TGF-β1/SMAD signalling is controlled at many stages besides ALK5-mediated R-SMAD phosphorylation. Especially the linker regions of SMAD2 and SMAD3 are targets of ALK5 independent phosphorylation via extracellular signal regulated kinases (ERK), mitogen activated kinases JNK and p38, glycogen synthetase kinase GSK as well as cyclin-dependent kinase CDK [50]. Phosphorylation via these kinases occurs downstream from TGFBR activation and they are known to modulate cellular TGF-β1 effects [50] and none of these mechanisms is shown to affect TJ localization of pSMAD2-C. However, this demonstrates ALK5-independent modulation of SMAD-mediated signalling and possibly the observed TJ accumulation of pSMAD2-C results from similar mechanisms.

The early stage of increase in paracellular permeability is characterized by the redistribution of CLDN3 from TJs into the nuclei. However, this is in contrast to pSMAD2-C that TJ localization remains almost non-affected by TGF-β1. Evidently, CLDN3 redistribution into the nucleus follows a mechanism that is independent from pSMAD2-C transport, even though ALK5 inhibitor A83-01 prevents not only the increase in paracellular permeability but also CLDN3 redistribution into the nucleus. This shows that both effects depend on ALK5 activation and hence on canonical TGF-β1 signalling pathways. Putative nuclear localization sequences (NLS) have been recognized in most claudins [18]. Nuclear CLDN1 localization was described for colon cancer cells and seems to modulate metastatic behaviour [13, 16] and also to be pivotal for aggressiveness of thyroid carcinoma [78]. CLDN2 that is not reported to contain putative NLS [18] enhances proliferative behaviour in lung adenocarcinoma [24]. A more recent study demonstrated nuclear localization of CLDN3 in colorectal adenocarcinoma [67]. Even though nuclear localization of several claudins seems to be associated with malignancy of several different tumours and can be often observed, it remained elusive how claudins enter the nucleus and what the functional consequence of their redistribution into the nucleus is. Our observation that TGF-β1 induces nuclear CLDN3 localization in bronchiolar epithelia demonstrates that nuclear CLDN localization is not solely linked to carcinoma cells and the herein investigated example points to a role for CLDN3 in TGF-β1 signalling in bronchial epithelia.

We showed that TGFBR1 and -R2 are expressed solely in ciliated but not in non-ciliated epithelial cells. Furthermore, pSMAD2-C accumulated at basal bodies of motile cilia shortly after TGF-β1 application. Again, the ALK5 inhibitor A83-01 abolished this initial effect. This clearly shows that the canonical TGF-β1 signalling pathway is activated and is needed to initiate TJ opening. Hence, ciliated cells are the direct targets of TGF-β1. Nonetheless, all epithelial cell types, ciliated as well as non-ciliated cells that do express neither TGFBR1 nor TGFBR2, showed a similar response to TGF-β1. In all cell types, we observe the redirection of CLDN3 from TJs into nuclei and the accumulation of nuclear pSMAD2-C. There was also no difference detectable in time course between TGF-β1-induced responses in ciliated versus non-ciliated cells. However, mechanisms that transduce the TGF-β1 signal from ciliated cells to non-ciliated cells could not be resolved. Possibly, paracrine mechanisms that depend on factors released by ciliated cells upon TGF-β1 exposure may account for the transduction. For instance, TGF-β1 has been shown to facilitate ATP release from lung cancer cells that potentiate cell motility by autocrine mechanisms [58, 59]. However, also TJ may serve as a signal transducing structure. Claudins establish trans-interactions with claudins of adjacent cells, and thereby they control paracellular permeability [21]. Those trans-interactions have been demonstrated also for CLDN3 with CLDN1, CLDN3 and CLDN5, and it has been demonstrated that the trans-interaction causes an enrichment of the trans-interacting claudin at the contact site in the membrane of the adjacent cells [48]. Probably removal of CLDN3 from TJ of ciliated cells triggers its redistribution in neighbouring cells to propagate TGF-β1 signal from the stimulated ciliated to contacting non-ciliated cells.

In summary, we demonstrated that TGF-β1 increases paracellular permeability of bronchial epithelia first via activating CLDN3 redistribution into the nucleus and finally inducing formation of epithelial lesions. These effects likely contribute to loss of epithelial integrity that has been described for atopic asthma patients [71]. Immune cells like macrophages and eosinophils secrete TGF-β1 into the airway surface liquid of conductive airways. Once TGF-β1 is released from its latency complex, it diffuses to its receptor. According to the gel-on-gel model [6], this diffusion is hindered due to the hydrogel character of the ASL. The localization of TGF-β1 receptors on motile cilia would facilitate the detection of active TGF-β1 ligands.

## Supplementary Information

ESM 1 (DOCX 24 kb).

ESM 2 (PDF 5457 kb).

ESM 3 (PDF 5594 kb).

ESM 4 (PDF 6498 kb).

ESM 5 (PDF 6047 kb).

ESM 6 (PDF 174 kb).

## Data Availability

Data and material will be available upon request.
