# Supplementary material for: TGF-β1 increases permeability of ciliated airway epithelia via redistribution of claudin 3 from tight junction into cell nuclei
Source: Pflugers Arch. 2021 Jan 2;473(2):287–311. doi: 10.1007/s00424-020-02501-2 (PMC7835204; doi:10.1007/s00424-020-02501-2)
Supplement: Supplementary file 1 — (DOCX 24 kb). [file 424_2020_2501_MOESM1_ESM.docx]

Supplementary information

Figure S1: Confocal images of primary human bronchial epithelial cells (hBEpC) also given within Figure 3A - C of the manuscript. Cells remained untreated as controls (CTRL). hBEpC were cultivated at ALI conditions for **A)** 19 days, **B)** 22 days and **C)** 28 days. Cells were immunostained for zonula occludens protein 1 as TJ counterstaining (ZO-1, red), Hoechst 3328 as nuclear counterstaining (Hoe, blue), pSMAD2-C (pSMAD2, green) and γ-tubulin (γTUB, green). Small insets at the left give grey scale images for each channel. RGB images represents merged channels (from left to right) all four channels, γTUB and pSMAD2, ZO-1 and pSMAD2, Hoe and pSMAD2. Schemes drawn on the left hand side of each row represents focus level (arrow). Upper row = focus level at the tight junctions, lower row = focus level at cell nuclei.

Figure S2: Confocal images of primary human bronchial epithelial cells (hBEpC) also given within Figure 3D - F of the manuscript. Cells were cultivated at ALI conditions until day 18 and subsequently treated with TGF-β1. **A)** For 1 day = TGF-β1 19 d, **B)** for 4 days = TGF-β1 22 d and **C)** for 10 days = TGF-β1 28 d. Cells were immunostained for zonula occludens protein 1 as TJ counterstaining (ZO-1, red), Hoechst 3328 as nuclear counterstaining (Hoe, blue), pSMAD2-C (pSMAD2, green) and γ-tubulin (γTUB, green). Small insets at the left give grey scale images for each channel. RGB images represents merged channels (from left to right) all four channels, γTUB and pSMAD2, ZO-1 and pSMAD2, Hoe and pSMAD2. Schemes drawn on the left hand side of each row represents focus level (arrow). Upper row = focus level at the tight junctioons, lower row = focus level at cell nuclei.

Figure S3: Confocal images of primary human bronchial epithelial cells (hBEpC) also given within Figure 5 A – C) of the manuscript. Cells remained untreated as controls (CTRL). hBEpC were cultivated at ALI conditions for **A)** 19 days, **B)** 22 days and **C)** 28 days. Cells were immunostained for zonula occludens protein 1 as TJ counterstaining (ZO-1, red), Hoechst 3328 as nuclear counterstaining (Hoe, blue), SMAD2 (SMAD2, green) and γ-tubulin (γTUB, cyan). Small insets at the left give grey scale images for each channel. RGB images represents merged channels (from left to right) all four channels, γTUB and SMAD2, ZO-1 and SMAD2, Hoe and SMAD2. Schemes drawn on the left hand side of each row represents focus level (arrow). Upper row = focus level at the tight junctions, lower row = focus level at cell nuclei.

Figure S4: Confocal images of primary human bronchial epithelial cells (hBEpC) also given within Figure 5 D – F**)** of the manuscript. Cells were cultivated at ALI conditions until day 18 and subsequently treated with TGF-β1. **A)** For 1 day = TGF-β1 19 d, **B)** for 4 days = TGF-β1 22 d and **C)** for 10 days = TGF-β1 28 d. Cells were immunostained for zonula occludens protein 1 as TJ counterstaining (ZO-1, red), Hoechst 3328 as nuclear counterstaining (Hoe, blue), SMAD2 (SMAD2, green) and γ-tubulin (γTUB, cyan). Small insets at the left give grey scale images for each channel. RGB images represents merged channels (from left to right) all four channels, γTUB and SMAD2, ZO-1 and SMAD2, Hoe and SMAD2. Schemes drawn on the left hand side of each row represents focus level (arrow). Upper row = focus level at the tight junctioons, lower row = focus level at cell nuclei.

Figure S5: Quantification of non-phosphorylated SMAD2 by western blot experiments. Protein abundance was performed on after western blotting and Immune detection of HSP90 proteins that served as internal calibrator and SMAD2 proteins. Western blot experiments of whole cell protein exptracts of control cells (CTRL) and TGF- β1 (TGF- β1) treated epithelia on **A)** cultivation day 19 that coresponds to 1 day TGF- β1 exposure and **B)** cultivation day 28 that coresponds to 10 days TGF- β1 exposur. Fluorescence intensity of SMAD2 staining was normalized to HSP90 fluorescence intensity. Box plots give SMAD2 protein abundance as relative arbitrary units (RAU) for control and TGF- β1 treated epithelia on **C)** cultivation day 19 and **D)** cultivation day 28. No difference in relative SMAD2 abundance between control and TGF- β1 treated cells were observed. All experiments N = 4 from 2 different donors)

Table S1

Transepithelial electrical resistance (TEER) as shown in Figure 1A. Comparision all vs TGFβ1, Kruskal-Wallis Test with Dunns Correction for multiple comparison

|  | TEER [Ωcm^2^] | |  |
| --- | --- | --- | --- |
|  | median | IQR | p-value / N |
| CTRL | 473.3 | 348.3 | <0.0001 / 18 |
| TGF-β1 | 17.5 | 43.1 |  |
| TGF-β1 + A83-01 | 699.7 | 461.2 | <0.0001 / 18 |

Table S2

Apparent permeability coefficients (P_app_) for sodium fluorescein (Na-fluo), 4 kDa and 20 kDa dextran (4 kDa and 20 kDa, respectively) in control (CTRL) and TGF- β1 (TGF- β1) treated epithelia on cultivation day 22 and 28 (4 days and 10 days of TGF- β1 exposure, respectively). Data are given as median and range as 25% and 75% quartile (25% / 75%). Statistical test CTRL vs TGF- β1: Mann-Whitney test (p / N)

|  |  | P_app_ [cm/s] CTRL | | P_app_ [cm/s], TGF-β1 | |  |
| --- | --- | --- | --- | --- | --- | --- |
|  |  | median | 25 % / 75% | median | 25 % / 75% | p-value / N |
| Day 22 | Na-fluo | 1.17e-6 | 7.92e-7 / 1.75e-6 | 6.59e-6 | 5.62e-6 / 7.12e-6 | <0.0001 / 8-9 |
|  | 4 kDa | 1.80e-7 | 4.86e-8 / 3.33e-7 | 1.81e-6 | 7.97e-7 / 6.35e-6 | =0.0003 / 9 |
|  | 20 kDa | 4.51e-8 | 7.11e-9 / 2.02e-7 | 3.69e-6 | 1.07e-7 / 4.04e-6 | =0.0056 / 9 |
| Day 28 | Na-fluo | 1.35e-6 | 8.17e-7 / 2.16e-6 | 6.43e-6 | 4.15e-6 7 6.63e-6 | <0.0001 / 9 |
|  | 4 kDa | 3.87e-7 | 1.50e-7 / 6.41e-7 | 6.04e-6 | 8.38e-7 / 1.03e-5 | <0.0001 / 9 |
|  | 20 kDa | 1.29e-7 | 2.38e-8 / 4.18e-7 | 3.53e-6 | 1.51e-6 / 6.68e-6 | <0.0001 / 9 |

Table S3

Transepithelial electrical resistance as shown in figure 1F. Data are given as median and range as 25% and 75% quartile (25% / 75%). Statistical test : ANOVA with Kruskal-Wallis post hoc test with Dunn’s correction for multiple comparison

|  | TEER[Ωcm^2^] | | |  | |
| --- | --- | --- | --- | --- | --- |
|  | median | 25 % / 75% | | N | vs / p-value |
| CTRL | 649.0 | | 572,5 / 1401 | 11 |  |
| TGF-β1 | 16.52 | | 12.27 / 28.29 | 12 | CTRL / <0.0001 |
| BMP2 | 255.3 | | 163,4 / 491.1 | 10 | CTRL / ns |
| Activin A | 422.2 | | 289.9 / 603.0 | 12 | CTRL / ns |
| TGF-β1 + A83-01 | 772.7 | | 575.7 / 1162 | 12 | TGF-β1 / <0.0001 |
| TGF-β1 + SB505124 | 542.5 | | 512.9 / 935.9 | 12 | TGF-β1 / <0.0001 |
| TGF-β1 + DMH-1 | 21.6 | | 6.823 / 44.46 | 12 | TGF-β1 / ns |
| TGF-β1 + LDN212854 | 15.08 | | 8.902 / 24.08 | 10 | TGF-β1 / ns |
| TGF-β1 + ML347 | 35.31 | | 23.16 / 166.0 | 12 | TGF-β1 / ns |
