# Supplementary figures and images for: TGF-β1 increases permeability of ciliated airway epithelia via redistribution of claudin 3 from tight junction into cell nuclei
Source: Pflugers Arch. 2021 Jan 2;473(2):287–311. doi: 10.1007/s00424-020-02501-2 (PMC7835204; doi:10.1007/s00424-020-02501-2)

# Figure S1

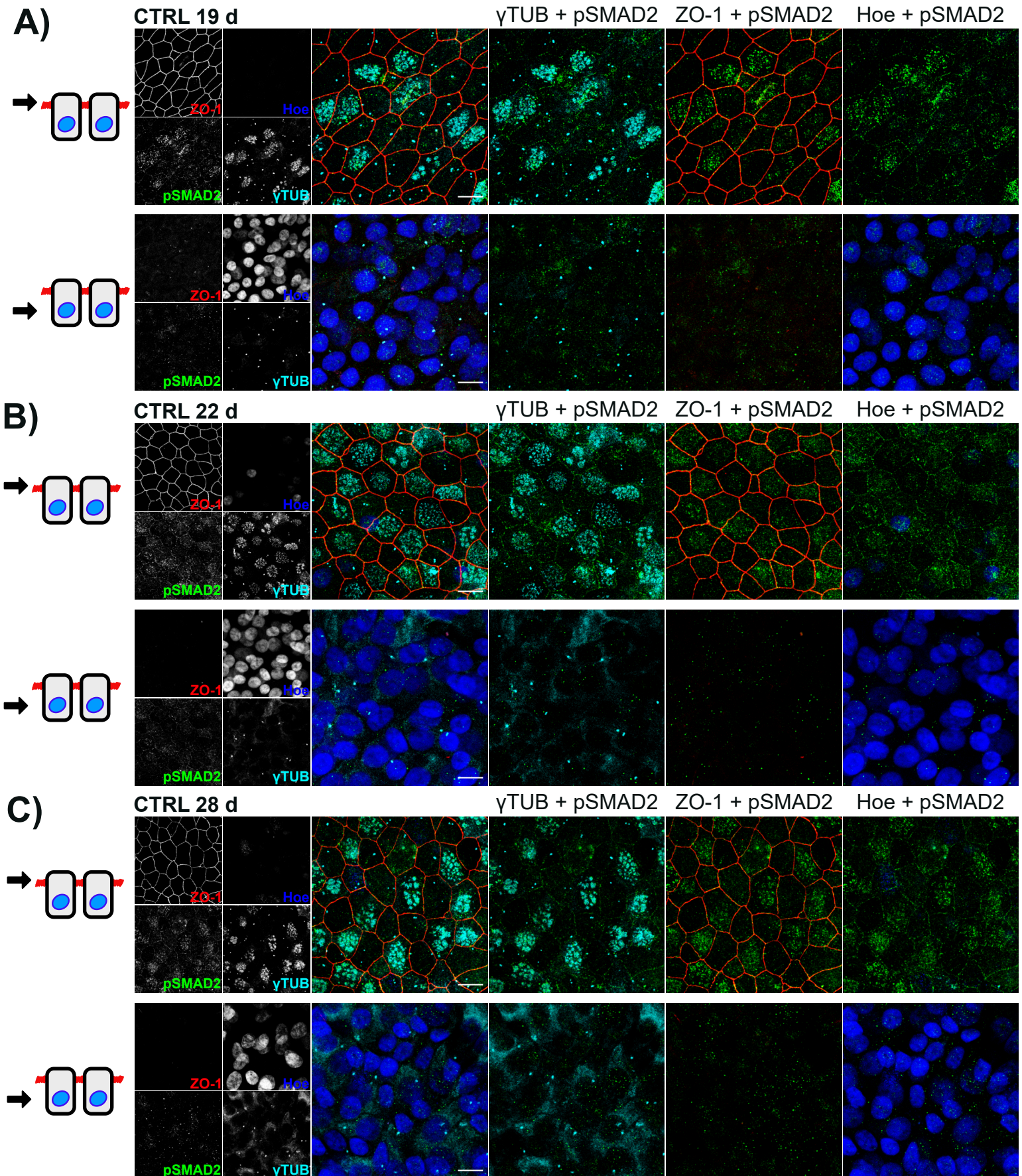

Supplement: Supplementary file 2 — (PDF 5457 kb). [file 424_2020_2501_MOESM2_ESM.pdf]

**Figure S2**

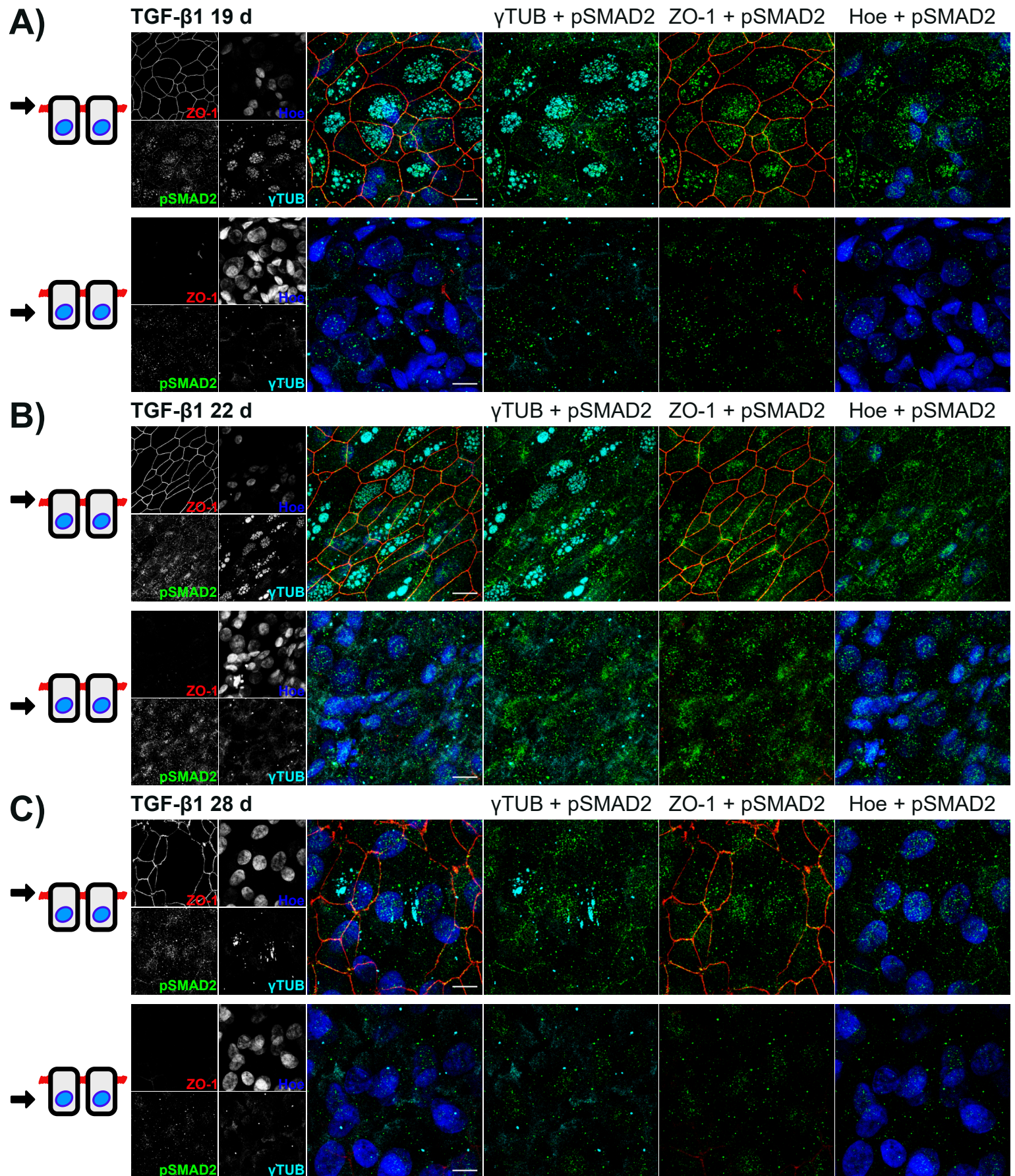

Supplement: Supplementary file 3 — (PDF 5594 kb). [file 424_2020_2501_MOESM3_ESM.pdf]

# Figure S3

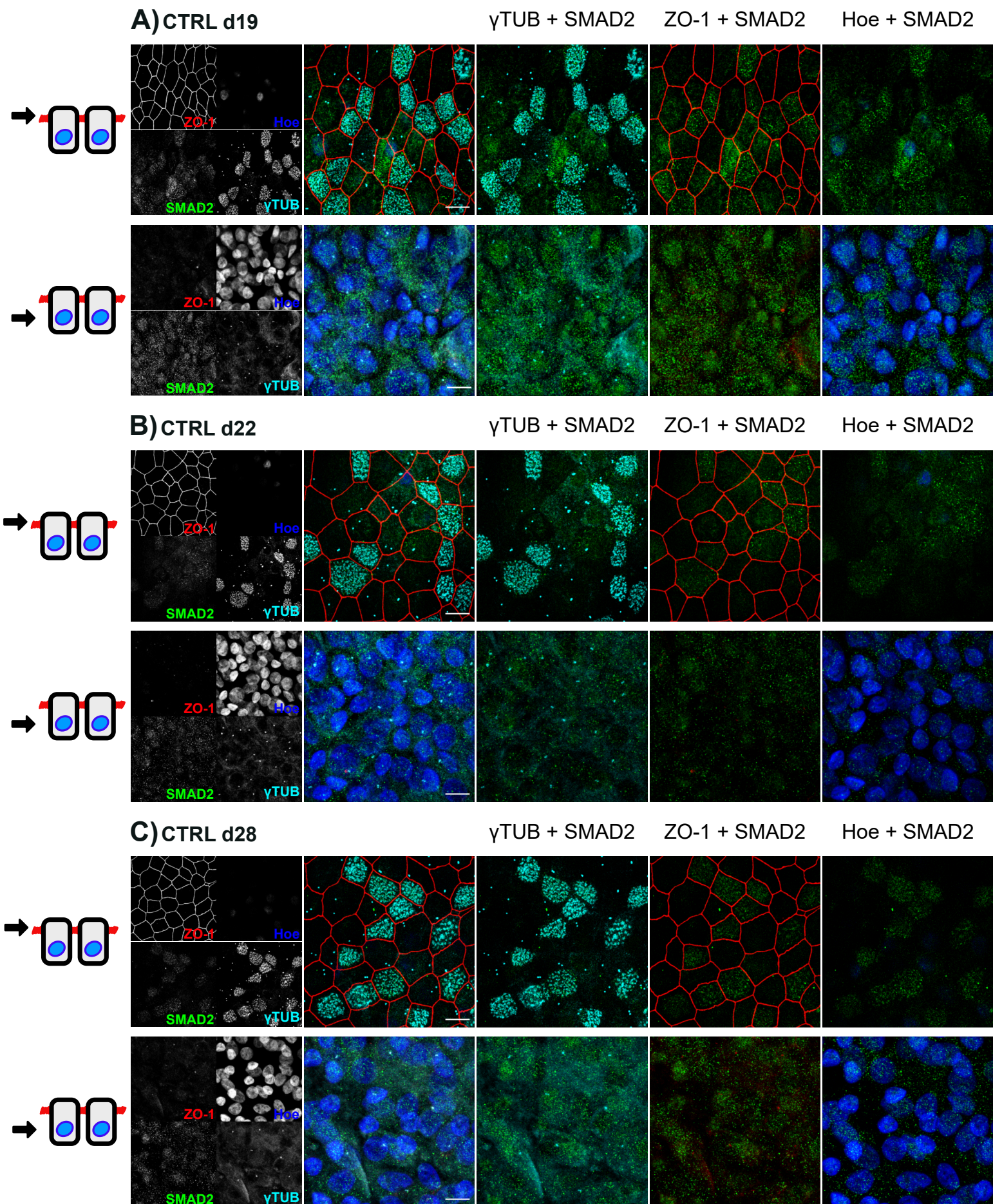

Supplement: Supplementary file 4 — (PDF 6498 kb). [file 424_2020_2501_MOESM4_ESM.pdf]

**Figure S5**

**A)** CTRL / TGF- $\beta$ 1 d19

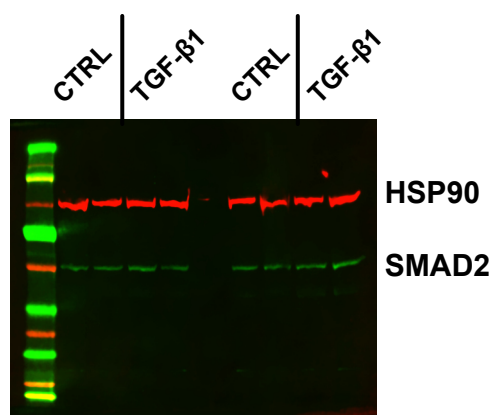

**B)** CTRL / TGF- $\beta$ 1 d28

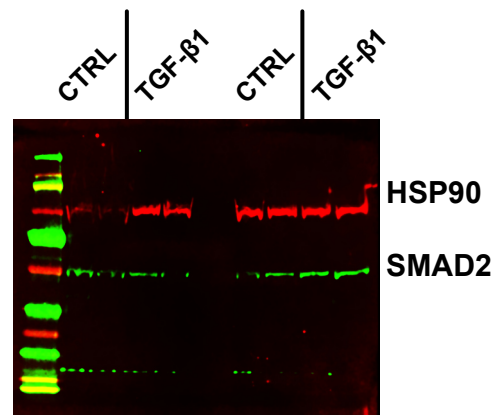

**C)**

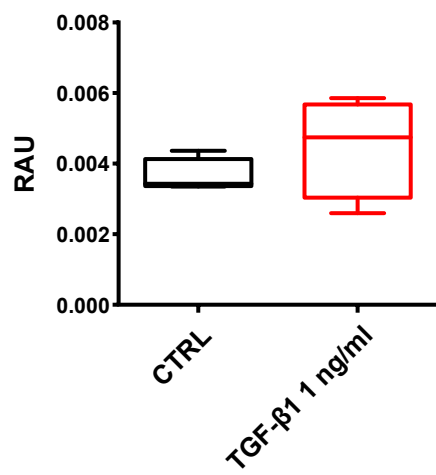

**D)**

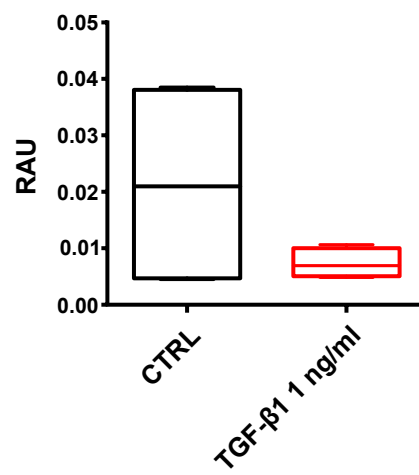

Supplement: Supplementary file 6 — (PDF 174 kb). [file 424_2020_2501_MOESM6_ESM.pdf]
