# Supplementary material for: TGF-β1 increases permeability of ciliated airway epithelia via redistribution of claudin 3 from tight junction into cell nuclei
Source: Pflugers Arch. 2021 Jan 2;473(2):287–311. doi: 10.1007/s00424-020-02501-2 (PMC7835204; doi:10.1007/s00424-020-02501-2)
Supplement: Supplementary file 5 — (PDF 6047 kb). [file 424_2020_2501_MOESM5_ESM.pdf]

Figure S4

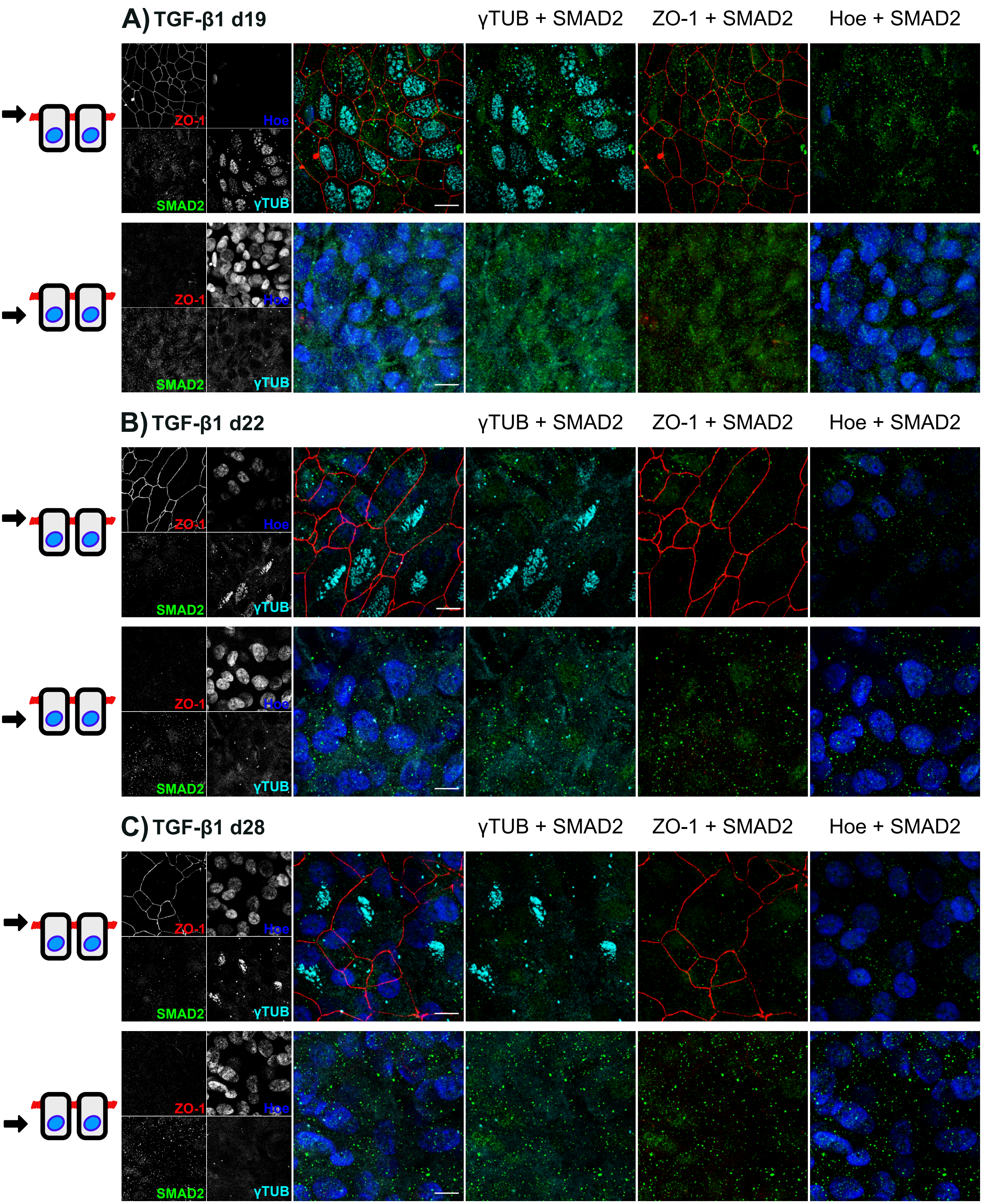
A) TGF-β1 d19
γTUB + SMAD2
ZO-1 + SMAD2
Hoe + SMAD2
ZO-1
Hoe
SMAD2
γTUB
B) TGF-β1 d22
C) TGF-β1 d28
